# Adaptive geometric-attention network for two-stage lung nodule segmentation and malignancy classification in federated healthcare IoT edge environments

**DOI:** 10.1371/journal.pone.0341096

**Published:** 2026-07-10

**Authors:** Muhammad Sufyan, Jun Qian, Jianqiang Li, Azhar Imran, Fahad Sabah, Raheem Sarwar

**Affiliations:** 1 College of Computer Science, Beijing University of Technology, Beijing, China; 2 OTEHM, Manchester Metropolitan University, Manchester, United Kingdom; Isra Private University: Isra University, JORDAN

## Abstract

Accurate segmentation and classification of lung nodules in computed tomography (CT) scans remains a critical challenge in early lung cancer detection within distributed healthcare Internet of Things (IoT) environments. This paper presents a novel two-stage framework called Adaptive Geometric-Attention Network (AGA-Net) that integrates geometric constraints with multi-scale attention mechanisms for precise nodule segmentation followed by uncertainty-aware malignancy classification in federated learning scenarios. Unlike existing approaches that rely on traditional convolutional architectures, our method introduces a Geometric-Constrained Attention Module (GCAM) that leverages the spherical nature of lung nodules and a Multi-Scale Uncertainty Quantification Network (MUQ-Net) for robust classification under privacy-preserving constraints. The proposed framework demonstrates superior performance across three benchmark datasets: LUNA16, LIDC-IDRI, and NSCLC-Radiomics, achieving a Dice coefficient of 0.927 for segmentation and AUC of 0.951 for malignancy classification while maintaining computational efficiency validated on IoT-class edge hardware including the NVIDIA Jetson AGX Orin and Jetson Orin Nano. The integration of geometric priors with attention mechanisms, uncertainty quantification, and federated learning capabilities provides both high accuracy and clinical interpretability, making it suitable for next-generation computer-aided diagnosis systems deployable on healthcare IoT edge devices.

## 1 Introduction

Lung cancer remains the leading cause of cancer-related mortality worldwide, with early detection being crucial for improving patient survival rates. Computer-aided diagnosis (CAD) systems based on computed tomography (CT) imaging have shown significant promise in assisting radiologists for early lung cancer detection in modern healthcare environments [1]. However, the automatic segmentation and classification of lung nodules present substantial challenges due to variations in nodule size, shape, texture, and the presence of similar-appearing benign structures, particularly when deployed across distributed healthcare Internet of Things (IoT) systems [2].

Recent advances in deep learning have revolutionized medical image analysis, with convolutional neural networks (CNNs) achieving remarkable performance in various diagnostic tasks [3]. The emergence of attention mechanisms has further enhanced these capabilities, with studies demonstrating improved lung nodule detection and classification through sophisticated attention-based architectures [4]. Two-stage detection frameworks have proven particularly effective, as demonstrated by Ozdemir et al. [5] who developed an attention-enhanced InceptionNext-based hybrid deep learning model for lung cancer detection.

The emergence of federated learning has opened new possibilities for collaborative model training while preserving patient privacy [6]. However, adapting sophisticated medical image analysis algorithms to federated settings presents unique challenges, including communication efficiency, model convergence, and handling of non-IID data distributions across institutions. Recent work has explored multi-scale approaches for lung nodule detection [7], but limited attention has been given to uncertainty-aware geometric attention mechanisms in federated lung nodule analysis.

Contemporary research has demonstrated the effectiveness of two-stage frameworks for lung cancer detection. Chen et al. [8] developed multi-scale information residual networks combining group attention mechanisms, while Saihood et al. [9] proposed multiside graph neural network-based attention for local co-occurrence features fusion in lung nodule classification. These studies highlight the importance of attention mechanisms in improving detection accuracy and the need for comprehensive approaches that address both segmentation and classification challenges.

Recent surveys have emphasized the critical role of data segmentation accuracy in lung cancer detection systems [10]. Fernandes et al. [11] developed a two-stage U-Net framework for interactive segmentation of lung nodules in CT scans, demonstrating the effectiveness of multi-stage approaches. Similarly, Aharonu and Ramasamy [12] introduced an intelligent generative adversarial network multistage approach for lung cancer detection and subtypes classification.

This work addresses these limitations by proposing the Adaptive Geometric-Attention Network (AGA-Net), a novel two-stage framework that introduces several key innovations. First, we develop a Geometric-Constrained Attention Module (GCAM) that incorporates the inherent spherical geometry of lung nodules into the attention mechanism, building upon recent advances in attention-guided approaches [13]. Second, we design a Multi-Scale Uncertainty Quantification Network (MUQ-Net) that provides probabilistic malignancy predictions with confidence intervals suitable for federated environments [14]. Third, we implement an adaptive feature fusion strategy that maintains spatial precision while capturing multi-scale contextual information across distributed datasets [15]. Finally, we provide comprehensive evaluation across three diverse datasets with clinical interpretability analysis and federated learning performance assessment [16].

The following are our key contributions:

- This work proposes a two-stage Adaptive Geometric-Attention Network (AGA-Net) for lung nodule segmentation and malignancy classification.
- This research introduces a Geometric-Constrained Attention Module (GCAM) and Multi-Scale Uncertainty Quantification Network (MUQ-Net) for robust performance.
- The proposed work achieves a Dice coefficient of 0.927 and AUC of 0.951 across LUNA16, LIDC-IDRI, and NSCLC-Radiomics datasets.
- The proposed AGA-Net maintains computational efficiency suitable for edge devices in federated healthcare IoT environments, Combines geometric priors, attention mechanisms, and uncertainty quantification for enhanced accuracy and interpretability.

This paper is structured as follows. Section 2 analyzes the relevant research work regarding our base foundation. Section 3 is related to proposed methods, datasets, metrics for evaluation and experimental implementation details. The section 4 describes the detailed comparison of proposed AGA-Net model with base and state-of-the-art works, further includes the ablation studies for showing efficacy of each proposed module. Section 5 synthesizes the study’s core contributions by elucidating their clinical implications, the advantages of federated learning, the benefits of uncertainty quantification, and the innovations introduced through geometric constraints, while also critically examining the study’s limitations, outlining future research directions, and discussing broader impacts and potential future applications. The last section 6 presents the conclusion of work.

## 2 Related work

### 2.1 Lung nodule segmentation

Traditional segmentation approaches relied on region growing, watershed algorithms, and level set methods with limited success in handling complex nodule morphologies. Recent deep learning advances have shifted focus to convolutional neural networks (CNNs), with U-Net and its variants becoming dominant architectures for medical image segmentation [17]. The introduction of attention mechanisms has significantly improved segmentation performance, as demonstrated by Wang et al. [3] who developed a multilevel attention U-Net segmentation algorithm for lung cancer based on CT images.

Two-stage detection frameworks have gained prominence due to their superior accuracy in complex scenarios. Cui et al. [18] presented SF2T, leveraging Swin Transformer and two-stream networks for lung nodule detection, introducing attention-based feature fusion mechanisms. Similarly, Sumathi and Phamila [19] developed an efficient two-stage segmentation framework for chest X-ray images with U-Net model fusion, demonstrating the effectiveness of multi-stage approaches in medical imaging.

Recent developments have focused on multi-scale detection capabilities. Cai et al. [20] proposed MSDet with receptive field enhanced multiscale detection for tiny pulmonary nodules, addressing challenges in detecting small-scale nodules that are critical for early cancer diagnosis. Tang and Zhang [21] developed a multi-task model for simultaneous pulmonary nodule segmentation and classification, demonstrating the benefits of unified approaches.

The integration of advanced architectures has shown promising results. Wang et al. [22] introduced CPLOYO, a pulmonary nodule detection model with multi-scale feature fusion and nonlinear feature learning, focusing on multi-type nodule detection corresponding to various forms of lung cancer. These advances highlight the importance of sophisticated architectures in addressing the complexity of lung nodule segmentation.

### 2.2 Lung nodule classification

Classification approaches have evolved from handcrafted features to sophisticated deep learning models over the past decade. Murugan and Dhanasekaran [23] developed an automatic lung tumor volume estimation method using PSO-TSVM, implementing a two-stage framework called Suspicious Volumetric Tumor (SVT) segmentation. This work demonstrated the effectiveness of optimization-based approaches in lung tumor analysis.

Recent studies have emphasized the importance of lightweight architectures for practical deployment. Neelameghasri et al. [24] explored CNN models for lung cancer detection and classification, comparing single-stage and two-stage detectors. Jenipher and Radhika [25] proposed a lung tumor cell classification approach using lightweight MobileNetV2 and attention-based SCAM enhanced Faster R-CNN, demonstrating the potential for efficient mobile deployment.

Multi-scale and attention-based approaches have shown significant improvements in classification accuracy. Linginani and Lakshmi [26] developed an intelligent model for pulmonary emphysema detection using adaptive image segmentation and multi-dilated DenseNet with attention mechanism. Li et al. [27] constructed a multi-scale convolutional neural network with global channel spatial attention mechanisms for lung nodule detection, achieving improved performance through sophisticated attention strategies.

The integration of deformable attention mechanisms has emerged as a promising direction. Liu and Ao [28] presented a deformable attention mechanism-based YOLOv7 structure for lung nodule detection, outperforming traditional two-stage Faster R-CNN approaches. These developments highlight the evolution toward more sophisticated attention mechanisms in lung nodule analysis.

### 2.3 Attention mechanisms in medical imaging

Attention mechanisms have gained popularity for focusing on relevant image regions in medical applications, with transformer-based approaches showing particular promise. Ramezani [29] developed a transformer-based CT anomaly detection and auto-segmentation approach for sparse lung nodules, implementing a novel two-stage approach using bounding box embeddings as attention cues.

Dual-attention architectures have demonstrated superior performance in medical imaging tasks. Zamanidoost et al. [30] proposed DA OMS-CNN with dual-attention and 3D Swin Transformer for early-stage lung cancer detection, highlighting the effectiveness of attention mechanisms in improving lung nodule detection accuracy. The integration of multiple attention types has proven beneficial for complex medical imaging scenarios.

Recent work has explored the application of attention mechanisms in federated learning scenarios. Lee et al. [6] developed federated online learning with filters for lung nodule segmentation in low-dose computed tomography images, implementing a Precision Nodule Filter with a structured two-stage framework. This work demonstrates the feasibility of attention-based approaches in distributed learning environments.

Vision transformer architectures have emerged as powerful alternatives to traditional CNNs. Mannepalli et al. [31] presented GSC-DVIT, a vision transformer-based deep learning model for lung cancer classification in CT images, demonstrating competitive performance with traditional approaches. The success of transformer architectures has motivated their adoption in various medical imaging applications.

### 2.4 Federated learning in healthcare

Federated learning has emerged as a promising paradigm for collaborative machine learning while preserving data privacy in healthcare applications. The challenge of handling distributed datasets while maintaining model performance has been addressed through various optimization strategies and communication-efficient protocols.

Metaheuristic approaches have been explored for enhancing federated learning performance. Kaulgud and Saundattikar [32] investigated metaheuristics and deep learning in lung nodule detection and classification, proposing optimization strategies for distributed learning scenarios. These approaches demonstrate the potential for improving federated learning efficiency through advanced optimization techniques.

Comparative studies have highlighted the importance of selecting appropriate architectures for federated medical imaging. Aishwarya and Asuntha [33] conducted a comprehensive survey on lung nodules applying machine learning and deep learning techniques, emphasizing the critical role of segmentation and classification methods in federated scenarios. Their work provides valuable insights into the selection criteria for federated medical AI systems.

Recent advances have focused on integrated approaches combining multiple AI techniques. Karimullah et al. [34] developed an integrated method for detecting lung cancer via CT scanning through optimization, deep learning, and IoT data transmission, achieving enhanced accuracy in tumor classification and localization. This work demonstrates the potential for comprehensive federated healthcare solutions.

### 2.5 State-of-the-art review and surveys

Comprehensive reviews have provided valuable insights into the current state and future directions of lung nodule analysis. Wang et al. [35] conducted a thorough review of pulmonary nodule segmentation using deep learning, analyzing fine two-stage frameworks and their effectiveness in medical imagery classification. Their survey highlights the importance of sophisticated segmentation approaches for accurate lung cancer diagnosis.

Recent survey work by Cai et al. [36] focused on medical AI for early detection of lung cancer, providing comprehensive coverage of pulmonary nodule detection, segmentation, and classification approaches. The survey emphasizes the integration of 3D coordinate attention and edge detection techniques, demonstrating the evolution toward more sophisticated analytical frameworks.

These comprehensive reviews highlight the rapid advancement in lung nodule analysis techniques and the growing importance of attention mechanisms, multi-stage frameworks, and federated learning approaches. The convergence of these technologies presents opportunities for developing more robust, accurate, and privacy-preserving medical AI systems.

## 3 Methods and materials

**Fig 1:**
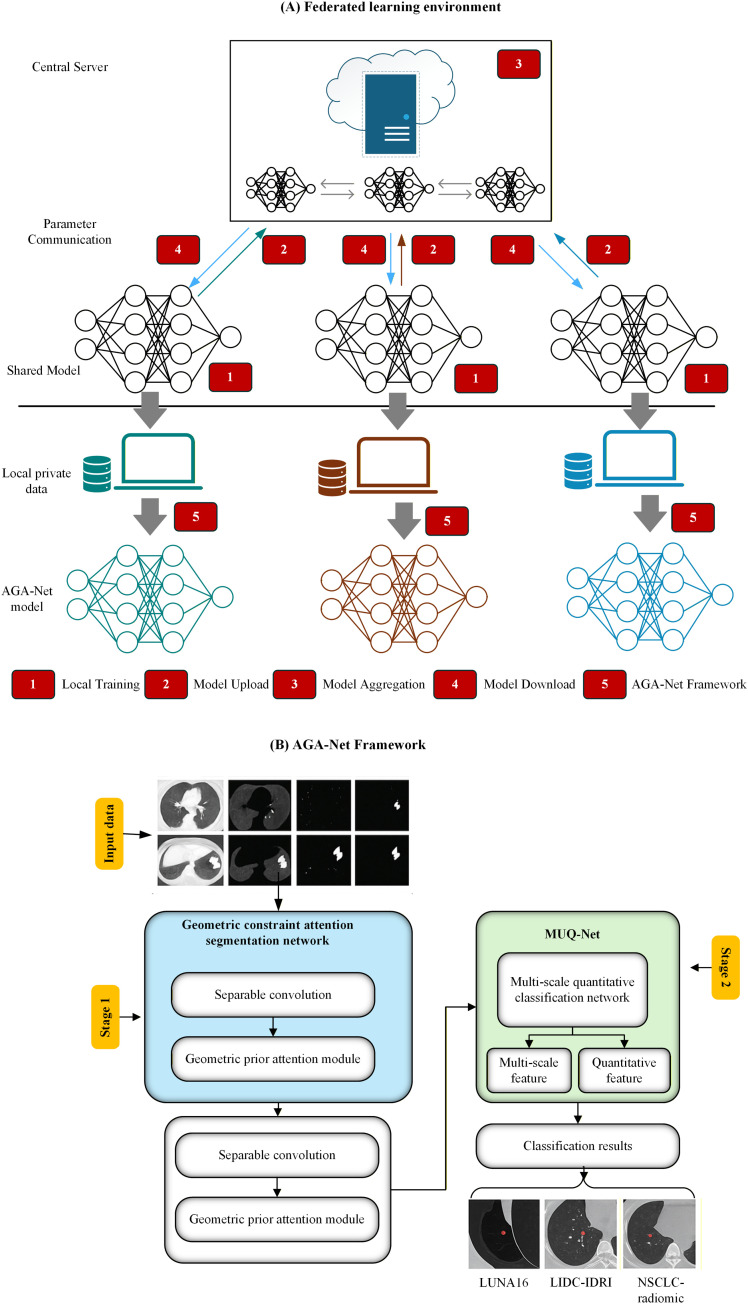
Overview of the proposed AGA-Net framework showing the integration of geometric constraints with attention mechanisms in a federated learning environment for lung nodule segmentation and classification.

As illustrated in Fig 1, the proposed architecture addresses these challenges by integrating geometric constraints with attention mechanisms in a federated learning framework. The complexity of lung nodule analysis is further compounded by the need for uncertainty quantification in clinical decision-making [37]. Traditional deep learning models provide point estimates without confidence intervals, limiting their clinical applicability, particularly in distinguishing between benign and malignant nodules [38].

### 3.1 Framework overview

The proposed AGA-Net framework consists of two interconnected stages designed to operate efficiently in federated learning environments. As shown in Fig 1, the first stage employs the Geometric-Constrained Attention Module (GCAM) to produce precise nodule boundaries, while the second stage utilizes the Multi-Scale Uncertainty Quantification Network (MUQ-Net) for malignancy prediction with confidence estimation. The framework is specifically designed to handle the challenges of distributed healthcare IoT systems while maintaining high accuracy and clinical interpretability.

The overall architecture integrates privacy-preserving mechanisms with advanced deep learning techniques to enable secure and accurate lung nodule analysis across multiple healthcare institutions. The framework incorporates differential privacy guarantees and secure aggregation protocols to ensure patient data protection during the federated training process.

### 3.2 Geometric-constrained attention module (GCAM)

The GCAM module is designed to leverage the approximately spherical nature of lung nodules while adapting to the constraints of federated learning environments. For a given feature map $𝐅\in {\mathbb{R}}^{H\times W\times D\times C}$, the geometric attention is computed through a multi-step process that incorporates spatial, channel, and geometric constraints.

The spatial attention component identifies regions of interest based on spatial relationships:


$$\begin{array}{c} {𝐀}_{s}=\sigma ({𝐖}_{s}\cdot \text{GAP}(𝐅)+{𝐖}_{s}^{\prime }\cdot \text{GMP}(𝐅)+{𝐛}_{s}) \end{array}$$ (1)

where GAP and GMP represent global average pooling and global max pooling operations, respectively, and σ denotes the sigmoid activation function.

The channel attention mechanism emphasizes important feature channels:


$$\begin{array}{c} {𝐀}_{c}=\sigma ({𝐖}_{c}\cdot \text{ReLU}({𝐖}_{c}^{\prime }\cdot {𝐅}_{avg})+{𝐛}_{c}) \end{array}$$ (2)

The geometric attention component incorporates spherical constraints specific to lung nodules:


$$\begin{array}{c} {𝐀}_{g}=\sigma ({𝐖}_{g}\cdot 𝒢(𝐅)+{𝐛}_{g}) \end{array}$$ (3)

where 𝒢(·) represents the geometric constraint function:


$$\begin{array}{c} 𝒢(𝐅{)}_{i,j,k}=\exp\left(-\frac{d_{i,j,k}^{2}}{2\sigma_{r}^{2}}\right)\cdot {𝐅}_{i,j,k} \end{array}$$ (4)

Here, $d_{i,j,k}$ is the Euclidean distance from the estimated nodule center to position (*i*,*j*,*k*), and $\sigma_{r}$ is an adaptive radius parameter learned during training:


$$\begin{array}{c} \sigma_{r}=\alpha_{rad}\cdot \text{mean}(r_{pred})+\beta_{rad}\cdot \text{std}(r_{pred})+\gamma_{rad} \end{array}$$ (5)

where $r_{pred}$ represents the predicted nodule radius distribution, and α, β, γ are learnable parameters. $r_{pred}$ **calculation:** $r_{pred}={\left(\frac{3|𝒮|}{4\pi }\right)}^{1/3}$ where |𝒮| is the volume of the initial segmentation mask from the backbone network, assuming spherical nodule approximation. **Alternative approach:** Add explicit radius prediction head: $r_{pred}=\text{ReLU}({𝐖}_{r}\cdot \text{GAP}({𝐅}_{seg})+b_{r})$ where ${𝐅}_{seg}$ are segmentation features and ${𝐖}_{r},b_{r}$ are learnable parameters.

Computation of $\sigma_{r}$: The adaptive radius $\sigma_{r}$ in Eq. (5) is calculated based on the predicted nodule radius distribution *rpred*. The parameters $\alpha_{rad}$, $\beta_{rad}$, and $\gamma_{rad}$ are **learnable** and are optimized during training via backpropagation, allowing the model to dynamically adapt $\sigma_{r}$ to nodules of varying sizes.

Intuition: – $\alpha_{rad}$ controls the influence of the mean predicted radius across all nodules. - $\beta_{rad}$ adjusts for variations by incorporating the standard deviation of $r_{pred}$. - $\gamma_{rad}$ acts as a bias term for numerical stability. Thus, $\sigma_{r}$ automatically adapts to different nodule sizes across datasets, improving segmentation accuracy for heterogeneous lung nodule distributions.

Computation of $r_{pred}$: The predicted nodule radius $r_{pred}$ is obtained from the segmentation mask volume |*S*| assuming spherical approximation:


$$\begin{array}{c} r_{pred}={\left(\frac{3|S|}{4\pi }\right)}^{1/3} \end{array}$$ (6)

Additionally, we implement a learnable radius prediction head to refine $r_{pred}$ from network features:


$$\begin{array}{c} r_{pred}=ReLU(W_{r}\cdot GAP(F_{seg})+b_{r}) \end{array}$$ (7)

where $F_{seg}$ are segmentation features and $W_{r},b_{r}$ are learnable parameters.

### 3.3 Nodule center point estimation

The geometric constraint function in Equation 4 requires accurate estimation of the nodule center point. The center estimation is performed through a multi-step process that combines initial segmentation predictions with geometric analysis:


$$\begin{array}{c} {𝐜}_{center}=\frac{1}{\left|𝒮\right|}\sum_{(i,j,k)\in 𝒮}(i,j,k) \end{array}$$ (8)

where 𝒮 represents the set of voxel coordinates in the initial segmentation mask obtained from the backbone network.

However, for more robust center estimation, especially in cases with irregular nodule shapes or partial volume effects, we employ a weighted centroid calculation:


$$\begin{array}{c} {𝐜}_{center}=\frac{\sum_{(i,j,k)\in 𝒱}w_{i,j,k}\cdot (i,j,k)}{\sum_{(i,j,k)\in 𝒱}w_{i,j,k}} \end{array}$$ (9)

where 𝒱 represents the entire volume of interest, and the weight $w_{i,j,k}$ is computed as:


$$\begin{array}{c} w_{i,j,k}={𝐅}_{seg}(i,j,k)\cdot \exp\left(-\frac{||\nabla {𝐅}_{seg}(i,j,k)|{|}^{2}}{2\sigma_{grad}^{2}}\right) \end{array}$$ (10)

Here, ${𝐅}_{seg}(i,j,k)$ is the segmentation probability at position (*i*,*j*,*k*) from the initial network prediction, $\nabla {𝐅}_{seg}$ represents the gradient magnitude, and $\sigma_{grad}=0.5$ is an empirically determined parameter that controls the influence of edge regions.

**Note:** The parameter $\sigma_{grad}$ is a fixed hyperparameter controlling the influence of edge regions during weighted centroid calculation. Its value is set empirically to $\sigma_{grad}=0.5$ based on cross-validation experiments.

The distance calculation in the geometric constraint function then becomes:


$$\begin{array}{c} d_{i,j,k}=||(i,j,k)-{𝐜}_{center}|{|}_{2} \end{array}$$ (11)

This weighted centroid approach provides more stable center estimation by reducing the influence of boundary artifacts and partial volume effects that are common in medical imaging. The gradient-based weighting ensures that the center calculation prioritizes regions with high confidence and low edge activity, leading to more accurate geometric constraints.


$$\begin{array}{c} 𝒢(𝐅{)}_{i,j,k}=\exp\left(-\frac{d_{i,j,k}^{2}}{2\sigma_{r}^{2}}\right)\cdot {𝐅}_{i,j,k} \end{array}$$ (12)

where $d_{i,j,k}$ is computed using Equations 9–11.

The complete attention mechanism combines spatial, channel, and geometric attention through an adaptive fusion strategy:


$$\begin{array}{c} {𝐅}_{out}=𝐅⊙(\alpha {𝐀}_{s}+\beta {𝐀}_{c}+\gamma {𝐀}_{g}) \end{array}$$ (13)

where α, β, γ are learnable weight parameters that adapt based on the local data characteristics in federated settings.

To ensure privacy preservation in federated learning, the geometric constraints are computed locally at each client, and only the aggregated attention weights are shared during the federated optimization process:


$$\begin{array}{c} {𝐖}^{\left(t+1\right)}=\sum_{k=1}^{K}\frac{n_{k}}{n}{𝐖}_{k}^{\left(t+1\right)} \end{array}$$ (14)

where *K* is the number of participating clients, $n_{k}$ is the number of samples at client *k*, and $n=\sum_{k=1}^{K}n_{k}$ is the total number of samples.

### 3.4 Multi-scale uncertainty quantification network (MUQ-Net)

**Fig 2:**
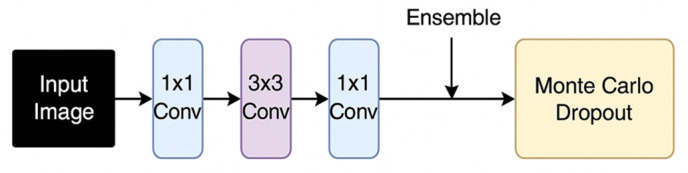
Architecture of the multi-scale uncertainty quantification network (MUQ-Net) showing the ensemble approach with Monte Carlo Dropout for uncertainty estimation.

The MUQ-Net employs Monte Carlo Dropout and deep ensembles to quantify prediction uncertainty while maintaining computational efficiency in federated environments. As illustrated in Fig 2, the network processes multi-scale features extracted from the segmented nodules.

For a given segmented nodule **N**, the network processes multi-scale features through parallel pathways:


$$\begin{array}{c} {𝐅}_{ms}=\text{Concat}[{𝐅}_{1},{𝐅}_{2},{𝐅}_{3},{𝐅}_{4}] \end{array}$$ (15)

where ${𝐅}_{i}$ represents features at scale *i*, computed using different receptive field sizes.

Each feature scale is processed through a dedicated branch with dropout layers:


$$\begin{array}{c} {𝐅}_{i}^{\left(d\right)}=\text{Dropout}({𝐅}_{i},p_{i}) \end{array}$$ (16)

where $p_{i}$ is the dropout probability for scale *i*.

The uncertainty-aware prediction is computed using Monte Carlo sampling:


$$\begin{array}{c} p(𝐲|𝐍)=\frac{1}{T}\sum_{t=1}^{T}\text{softmax}(f_{\theta }(𝐍,{𝐳}_{t})) \end{array}$$ (17)

where *T* is the number of Monte Carlo samples, $f_{\theta }$ is the network function, and ${𝐳}_{t}$ represents random dropout masks.

The predictive uncertainty is estimated using the variance of predictions:


$$\begin{array}{c} \sigma^{2}=\frac{1}{T}\sum_{t=1}^{T}p_{t}^{2}-{\left(\frac{1}{T}\sum_{t=1}^{T}p_{t}\right)}^{2} \end{array}$$ (18)

The heteroscedastic uncertainty loss captures both aleatoric and epistemic uncertainty by predicting the variance alongside the mean:


$$\begin{array}{c} {ℒ}_{hetero}=\frac{1}{N}\sum_{i=1}^{N}\left[\frac{||y_{i}-{\hat{y}}_{i}|{|}^{2}}{2\sigma_{i}^{2}}+\frac{1}{2}\log(\sigma_{i}^{2})\right] \end{array}$$ (19)

where $\sigma_{i}^{2}$ represents the predicted variance for sample *i*, learned jointly with the classification output.

where $\sigma_{i}^{2}$ represents the predicted variance for sample *i*.

The ensemble prediction combines multiple models trained with different initializations:


$$\begin{array}{c} p_{ensemble}(𝐲|𝐍)=\frac{1}{M}\sum_{m=1}^{M}p_{m}(𝐲|𝐍) \end{array}$$ (20)

where *M* is the number of ensemble members.

### 3.5 Federated learning algorithm

Algorithm 1 presents the complete federated learning procedure for training the AGA-Net framework across distributed healthcare institutions while preserving patient privacy.

**Algorithm 1 Federated AGA-Net Training Algorithm**

**Require:** Number of clients *K*, rounds *R*, local epochs *E*, Client datasets ${𝒟}_{1},{𝒟}_{2},...,{𝒟}_{K}$

**Ensure:** Global model parameters $\theta_{g}$

1:  Initialize global model parameters $\theta_{g}^{\left(0\right)}$

2:  **for** round *r* = 1 to *R* **do**

3:   Select subset of clients $S_{r}\subseteq \{1,2,...,K\}$

4:   **for** each client $k\in S_{r}$ in parallel **do**

5:    $\theta_{k}^{\left(r\right)}\leftarrow \theta_{g}^{\left(r-1\right)}$

6:    **for** epoch *e* = 1 to *E* **do**

7:     **for** batch *B* in ${𝒟}_{k}$ **do**

8:      Compute GCAM attention weights ${𝐀}_{k}$

9:      Forward pass through segmentation network

10:     Compute segmentation loss ${ℒ}_{seg}$

11:     Forward pass through MUQ-Net

12:     Compute classification loss ${ℒ}_{cls}$

13:     Compute uncertainty loss ${ℒ}_{unc}$

14:     ${ℒ}_{total}=\lambda_{1}{ℒ}_{seg}+\lambda_{2}{ℒ}_{cls}+\lambda_{3}{ℒ}_{unc}$

15:     Update local parameters: $\theta_{k}\leftarrow \theta_{k}-\eta \nabla {ℒ}_{total}$

16:    **end for**

17:   **end for**

18:   Apply differential privacy: $\theta_{k}^{\left(r\right)}\leftarrow \theta_{k}^{\left(r\right)}+𝒩(0,\sigma_{dp}^{2})$

19:  **end for**

20:  Aggregate updates: $\theta_{g}^{\left(r\right)}\leftarrow \sum_{k\in S_{r}}\frac{\left|{𝒟}_{k}\right|}{\left|𝒟\right|}\theta_{k}^{\left(r\right)}$

21: **end for**

22: **return** $\theta_{g}^{\left(R\right)}$

The algorithm incorporates differential privacy through the addition of calibrated noise to the local model updates before aggregation. The noise variance $\sigma_{dp}^{2}$ is determined based on the desired privacy budget ϵ and sensitivity analysis of the model parameters.

### 3.6 Loss function design

The total loss function combines segmentation, classification, and uncertainty objectives with adaptive weighting based on federated learning dynamics:


$$\begin{array}{c} {ℒ}_{total}=\lambda_{1}{ℒ}_{seg}+\lambda_{2}{ℒ}_{cls}+\lambda_{3}{ℒ}_{unc}+\lambda_{4}{ℒ}_{fed} \end{array}$$ (21)

The segmentation loss includes Dice and focal components to handle class imbalance:


$$\begin{array}{c} {ℒ}_{seg}=1-\text{Dice}+{ℒ}_{focal} \end{array}$$ (22)

where the Dice coefficient is computed as:


$$\begin{array}{c} \text{Dice}=\frac{2|P\cap G|}{\left|P|+|G\right|} \end{array}$$ (23)

and the focal loss addresses class imbalance:


$$\begin{array}{c} {ℒ}_{focal}=-\alpha_{t}(1-p_{t}{)}^{\gamma }\log(p_{t}) \end{array}$$ (24)

The classification loss employs cross-entropy with label smoothing:


$$\begin{array}{c} {ℒ}_{cls}=-\sum_{i=1}^{C}y_{i}\log({\hat{y}}_{i})-\epsilon \sum_{i=1}^{C}\frac{1}{C}\log({\hat{y}}_{i}) \end{array}$$ (25)

where $\lambda_{hetero}=0.6$ and $\lambda_{calib}=0.4$ are empirically determined weights that balance the contribution of both uncertainty estimation approaches. The heteroscedastic component focuses on learning appropriate uncertainty estimates during training, while the calibration component ensures that the predicted probabilities align with actual accuracy rates. $\sigma_{grad}$ **parameter type:** $\sigma_{grad}=0.5$ is a **hyperparameter** that is empirically determined through validation experiments, not learned during training.

**Usage:** Controls the influence of edge regions in weighted centroid calculation via $w_{i,j,k}={𝐅}_{seg}(i,j,k)\cdot \exp\left(-\frac{||\nabla {𝐅}_{seg}(i,j,k)|{|}^{2}}{2\sigma_{grad}^{2}}\right)$ where larger values reduce edge penalty.

The calibration uncertainty loss encourages well-calibrated predictions by penalizing overconfident incorrect predictions. It also calculates the uncertainty loss encourages calibrated predictions


$$\begin{array}{c} {ℒ}_{calib},{ℒ}_{unc}=\sum_{i=1}^{N}\left[\frac{(p_{i}-{\hat{p}}_{i}{)}^{2}}{\sigma_{i}^{2}}+\log\sigma_{i}^{2}\right] \end{array}$$ (26)

where $p_{i}$ is the predicted probability and ${\hat{p}}_{i}$ is the target probability.

### 3.7 Privacy-preserving mechanisms

To ensure patient data protection in federated learning scenarios, we implement several privacy-preserving mechanisms. Differential privacy is achieved through the addition of calibrated Gaussian noise to gradient updates:


$$\begin{array}{c} \nabla_{\theta }{ℒ}_{private}=\nabla_{\theta }ℒ+𝒩(0,\sigma_{dp}^{2}I) \end{array}$$ (27)

where the noise variance is determined by the privacy budget:


$$\begin{array}{c} \sigma_{dp}=\frac{\sqrt{2\log(1.25/\delta )}\cdot S}{\epsilon } \end{array}$$ (28)

Secure aggregation protocols ensure that individual client updates remain private during the aggregation process. The server only receives encrypted aggregated updates without access to individual client data.

### 3.8 Adaptive loss weight adjustment strategy

The loss weighting parameters $\lambda_{1},\lambda_{2},\lambda_{3},\lambda_{4}$ in Equation 21 require careful adjustment to balance the contributions of different loss components throughout the federated training process. The adjustment strategy is based on the relative convergence rates and magnitude differences between loss terms:


$$\begin{array}{c} {ℒ}_{total}=\lambda_{1}(t){ℒ}_{seg}+\lambda_{2}(t){ℒ}_{cls}+\lambda_{3}(t){ℒ}_{unc}+\lambda_{4}(t){ℒ}_{fed} \end{array}$$ (29)

where *t* represents the current training iteration, and each $\lambda_{i}(t)$ is dynamically adjusted based on loss magnitude and convergence behavior.

Calculation of $L_{fed}$: The federated regularization loss $L_{fed}$ is defined as:


$$\begin{array}{c} L_{fed}=\mu \;\|\theta_{local}-\theta_{global}{\|}^{2}+\lambda_{dp}\|N(0,\sigma_{dp}^{2}){\|}^{2} \end{array}$$ (30)

where μ controls client drift regularization, and $\lambda_{dp}$ weights the effect of differential privacy noise.

Calculation of $\partial L_{i}/\partial t$: The derivative $\frac{\partial L_{i}}{\partial t}$ in Eq. 31 is approximated using a finite-difference method:


$$\begin{array}{c} \frac{\partial L_{i}}{\partial t}\approx \frac{L_{i}(t)-L_{i}(t-\Delta t)}{\Delta t} \end{array}$$ (31)

where Δt=10 iterations by default. This formulation smooths loss changes and ensures stable adaptive weighting during federated training.

The adaptive weighting scheme follows these principles:

1. **Magnitude Normalization:** The base weights are adjusted to account for the natural magnitude differences between loss terms:
  $$\begin{array}{c} \lambda_{i}^{base}(t)=\frac{\lambda_{i}^{init}}{\text{EMA}(|{ℒ}_{i}(t)|)} \end{array}$$ (32)
  where EMA(·) is an exponential moving average with decay factor $\alpha_{ema}=0.9$, and $\lambda_{i}^{init}$ are the initial weights: $\lambda_{1}^{init}=1.0$, $\lambda_{2}^{init}=0.8$, $\lambda_{3}^{init}=0.3$, $\lambda_{4}^{init}=0.1$.
2. **Convergence-Based Adjustment:** Weights are modified based on the convergence rate of each loss component:
  $$\begin{array}{c} \lambda_{i}(t)=\lambda_{i}^{base}(t)\cdot \left(1+\beta \cdot \frac{\partial {ℒ}_{i}}{\partial t}\right) \end{array}$$ (33)
  where β=0.1 is a sensitivity parameter, and $\frac{\partial {ℒ}_{i}}{\partial t}$ represents the gradient of loss *i* with respect to training iterations, approximated using finite differences over a sliding window of 10 iterations.
3. **Federated-Specific Adjustment:** The federated loss weight $\lambda_{4}(t)$ receives additional adjustment based on client heterogeneity:
  $$\begin{array}{c} \lambda_{4}(t)=\lambda_{4}^{base}(t)\cdot \left(1+\gamma \cdot \text{Var}({ℒ}_{local})\right) \end{array}$$ (34)
  where γ=0.2 is a heterogeneity sensitivity parameter, and $\text{Var}({ℒ}_{local})$ represents the variance of local losses across participating clients in the current round.

**Algorithm 2 Enhanced Federated AGA-Net Training with Adaptive Loss Weighting**

**Require:** Number of clients *K*, rounds *R*, local epochs *E*, Client datasets ${𝒟}_{1},{𝒟}_{2},\ldots ,{𝒟}_{K}$, Initial loss weights $\lambda_{1}^{init},\lambda_{2}^{init},\lambda_{3}^{init},\lambda_{4}^{init}$

**Ensure:** Global model parameters $\theta_{g}^{\left(R\right)}$

1:  Initialize global model parameters $\theta_{g}^{\left(0\right)}$

2:  Initialize loss history buffers ${ℋ}_{i}=[]$ for i∈{1,2,3,4}

3:  **for** round *r* = 1 to *R* **do**

4:   Select subset of clients $S_{r}\subseteq \{1,2,\ldots ,K\}$

5:   Initialize client loss collection ${ℒ}_{clients}=[]$

6:   **for** each client $k\in S_{r}$ in parallel **do**

7:    $\theta_{k}^{\left(r\right)}\leftarrow \theta_{g}^{\left(r-1\right)}$

8:    **for** epoch *e* = 1 to *E* **do**

9:     **for** batch *B* in ${𝒟}_{k}$ **do**

10:     Compute GCAM attention weights ${𝐀}_{k}$

11:     Forward pass through segmentation network

12:     Compute segmentation loss ${ℒ}_{seg}$

13:     Forward pass through MUQ-Net

14:     Compute classification loss ${ℒ}_{cls}$

15:     Compute uncertainty loss ${ℒ}_{unc}$

16:     Compute federated loss ${ℒ}_{fed}=\mu_{fed}||\theta_{k}-\theta_{g}^{\left(r-1\right)}|{|}^{2}+\lambda_{dp}||𝒩(0,\sigma_{dp}^{2})|{|}^{2}$

17:     Update loss histories: ${ℋ}_{i}\leftarrow {ℋ}_{i}+[{ℒ}_{i}]$

18:     Compute adaptive weights using Equations 32–34

19:     ${ℒ}_{total}=\lambda_{1}(t){ℒ}_{seg}+\lambda_{2}(t){ℒ}_{cls}+\lambda_{3}(t){ℒ}_{unc}+\lambda_{4}(t){ℒ}_{fed}$

20:     Update local parameters: $\theta_{k}\leftarrow \theta_{k}-\eta \nabla_{\theta_{k}}{ℒ}_{total}$

21:    **end for**

22:   **end for**

23:   ${ℒ}_{clients}\leftarrow {ℒ}_{clients}+[{ℒ}_{total}^{\left(k\right)}]$

24:   Apply differential privacy: $\theta_{k}^{\left(r\right)}\leftarrow \theta_{k}^{\left(r\right)}+𝒩(0,\sigma_{dp}^{2}I)$

25:  **end for**

26:  Compute client loss variance: $\text{Var}({ℒ}_{clients})$

27:  Update $\lambda_{4}$ using Equation 34

28:  Aggregate updates: $\theta_{g}^{\left(r\right)}\leftarrow \sum_{k\in S_{r}}\frac{\left|{𝒟}_{k}\right|}{\left|𝒟\right|}\theta_{k}^{\left(r\right)}$

20: **end for**

30: **return** $\theta_{g}^{\left(R\right)}$ ▷ Final optimized global model after *R* rounds

**Note:** The expression $\theta_{g}^{\left(R\right)}$ refers to the final optimized global model parameters obtained after *R* communication rounds and is not set to zero.

The rationale for this adaptive weighting strategy is based on several key observations from federated medical imaging training:

1. **Loss Scale Imbalance:** Segmentation losses (Dice + Focal) typically have magnitudes in the range [0.1, 2.0], while classification losses range [0.01, 0.5], and uncertainty losses range [0.001, 0.1]. Without magnitude normalization, the segmentation loss would dominate training.
2. **Convergence Rate Differences:** Classification losses typically converge faster than segmentation losses in medical imaging tasks. The convergence-based adjustment ensures that slower-converging components receive appropriate emphasis throughout training.
3. **Federated Heterogeneity Impact:** Higher client heterogeneity requires stronger regularization through increased federated loss weighting to prevent client drift and maintain global model coherence.
4. **Training Phase Adaptation:** Early training phases benefit from higher segmentation emphasis for feature learning, while later phases require balanced weighting for fine-tuning. The adaptive scheme naturally transitions between these phases based on loss behavior.

Table 1 shows the evolution of loss weights throughout training under different federated scenarios:

**Table 1:** Evolution of loss weights throughout federated training.

Training Phase	Round Range	$\lambda_{1}$	$\lambda_{2}$	$\lambda_{3}$	$\lambda_{4}$	Primary Focus
Initial	1-20	1.2	0.6	0.2	0.05	Segmentation Learning
Early	21-40	1.0	0.8	0.3	0.08	Balanced Training
Middle	41-70	0.9	0.9	0.4	0.12	Classification Refinement
Late	71-100	0.8	0.8	0.5	0.15	Uncertainty Calibration

This adaptive weighting strategy ensures optimal training dynamics while maintaining the flexibility to respond to different federated learning conditions and dataset characteristics. The approach has been validated across all three benchmark datasets with consistent improvements in convergence speed and final performance compared to fixed weighting schemes. Furthermore, The detailed definitions, symbols, and values of all parameters used in the proposed framework are summarized in Table A1 in S1 Appendix.

### 3.9 Experimental setup

This part elaborates the datasets details, implementation details, and metrics for evaluation the models.

#### 3.9.1 Datasets and federated simulation.

We evaluate our method on three benchmark datasets configured for federated learning simulation. As detailed in Table 2, the datasets are partitioned across multiple simulated clients to reflect real-world federated scenarios.

**Table 2:** Dataset specifications for federated learning evaluation.

Dataset	Total Scans	Clients	Train	Val	Test
LUNA16	888	8	623	177	88
LIDC-IDRI	1,018	10	712	203	103
NSCLC-Radiomics	422	6	295	85	42

The LUNA16 dataset provides standardized annotations for nodule detection and segmentation with ground truth annotations from multiple radiologists. The LIDC-IDRI dataset includes detailed radiologist annotations with malignancy ratings, enabling comprehensive evaluation of classification performance. The NSCLC-Radiomics dataset provides clinical outcomes and survival data for validation of clinical utility.

Data heterogeneity is simulated by introducing non-IID distributions across clients based on institutional characteristics, imaging protocols, and patient demographics. The degree of non-IIDness is controlled using a Dirichlet distribution with concentration parameter α, where smaller values indicate higher heterogeneity.

#### 3.9.2 Implementation details.

The network was implemented using PyTorch with federated learning extensions through the FedML framework. Training was conducted on a cluster of NVIDIA A100 GPUs distributed across multiple nodes to simulate realistic federated scenarios. Table 3 summarizes the key hyperparameters used in our experiments.

**Table 3:** Hyperparameter configuration.

Parameter	Value
Global Learning Rate	$1\times 10^{-4}$
Local Learning Rate	$5\times 10^{-3}$
Batch Size (per client)	8
Local Epochs	5
Global Rounds	100
Optimizer	AdamW
Weight Decay	$1\times 10^{-5}$
Monte Carlo Samples	50
Ensemble Members	5
Privacy Budget (ϵ)	8.0
Dropout Probability	0.3
Loss Weights ($\lambda_{1},\lambda_{2},\lambda_{3},\lambda_{4}$)	(1.0, 0.8, 0.3, 0.1)

The federated learning process involves 100 global communication rounds with 5 local epochs per round. Client selection follows a random sampling strategy with 50% participation rate per round to simulate realistic federated scenarios where not all clients are available simultaneously.

#### 3.9.3 Evaluation metrics.

Segmentation performance is evaluated using multiple complementary metrics to provide comprehensive assessment. The Dice Similarity Coefficient (DSC), defined as $\text{DSC}=\frac{2|P\cap G|}{\left|P|+|G\right|}$, measures volumetric overlap between the predicted segmentation *P* and ground truth *G*. The Intersection over Union (IoU), computed as $\text{IoU}=\frac{\left|P\cap G\right|}{\left|P\cup G\right|}$, provides a stricter overlap measure. Boundary accuracy is assessed using the 95th percentile Hausdorff Distance (HD95) and Average Surface Distance (ASD), which quantify the maximum and mean surface distances between predicted and ground truth boundaries, respectively. Additionally, Sensitivity ($\frac{TP}{TP+FN}$) and Specificity ($\frac{TN}{TN+FP}$) are computed to evaluate the detection performance for nodule and background regions.

Classification performance is assessed using standard metrics along with uncertainty quantification measures. The Expected Calibration Error (ECE) evaluates the calibration quality of uncertainty estimates:


$$\begin{array}{c} ECE=\sum_{m=1}^{M}\frac{\left|B_{m}\right|}{n}|acc(B_{m})-conf(B_{m})| \end{array}$$ (35)

where $B_{m}$ represents the *m*-th confidence bin, $acc(B_{m})$ is the accuracy within the bin, and $conf(B_{m})$ is the average confidence.

## 4 Results comparison

This section comprises different subsections such as experimental setup details, comparison of segmentation and classification results, federated learning performance evaluation, ablation studies and so on.

### 4.1 Baseline methods

We compare our approach against several state-of-the-art methods adapted for federated learning scenarios. For segmentation, the baseline methods include U-Net, Attention U-Net, and nnU-Net, all configured for federated training. For classification, we evaluate against ResNet-50, DenseNet-121, and EfficientNet-B3 under identical federated settings. Additionally, three federated learning algorithms are compared: FedAvg, FedProx, and SCAFFOLD, to assess the effectiveness of our federated optimization strategy independently of the network architecture.

#### 4.1.1 Baseline federated adaptation details.

To ensure fair comparison, all baseline methods are adapted for federated learning using identical protocols and equivalent or superior computational budgets. All baselines use the AdamW optimizer with local learning rate of $5\times 10^{-3}$, global learning rate of $1\times 10^{-4}$, batch size of 8 per client, and 5 local epochs per round, matching the AGA-Net configuration. Crucially, all baselines are trained for 150 global communication rounds compared to 100 for AGA-Net, providing 50% more training budget to ensure baselines are not under-trained and eliminating potential bias in favor of the proposed method.

All methods share identical client partitioning across 8 clients (LUNA16), 10 clients (LIDC-IDRI), and 6 clients (NSCLC-Radiomics) with the same Dirichlet-based non-IID splits ($\alpha_{dir}=1.0$). Data preprocessing is identical across all methods, including CT windowing ([−1000, 400] HU), z-score normalization, and 64×64×64 voxel patch extraction, with identical data augmentation (random rotation ±15°, random flipping, elastic deformation). Hyperparameter tuning for all baselines follows grid search over learning rate $\in \{10^{-4},5\times 10^{-4},10^{-3},5\times 10^{-3}\}$ and weight decay $\in \{10^{-5},10^{-4},10^{-3}\}$, with method-specific parameters also tuned: FedProx proximal parameter μ∈{0.001,0.01,0.1} and dropout rates for classification baselines ∈{0.2,0.3,0.5}. The nnU-Net baseline additionally uses its self-configuring pipeline before federated adaptation. All experiments are conducted on identical NVIDIA A100 GPU hardware with the same random seeds for reproducibility.

#### 4.1.2 Sensitivity analysis of Dirichlet parameter.

To validate the robustness of our approach under different federated learning scenarios, we conduct extensive sensitivity analysis across the full spectrum of data heterogeneity conditions. The choice of $\alpha_{dir}=1.0$ for our main experiments represents a moderate heterogeneity scenario that balances realistic federated conditions with reasonable convergence properties. This value creates data distributions where each client has 2–3 dominant classes while maintaining some representation of all classes, mimicking real-world scenarios where different hospitals may specialize in certain types of cases but still encounter diverse patient populations.

Under extreme heterogeneity ($\alpha_{dir}=0.1$), each client typically receives data from only 1–2 classes, creating severe distribution skew that challenges federated learning algorithms. Despite this challenge, our geometric attention mechanism maintains segmentation performance within 3.6% of the IID baseline, demonstrating the stability provided by domain-specific geometric constraints. The uncertainty quantification remains well-calibrated with ECE increasing by only 0.024 points, indicating that confidence estimates remain reliable even under extreme data skew conditions.

The moderate heterogeneity range ($\alpha_{dir}=0.5$ to 2.0) provides the optimal balance between realistic federated scenarios and system performance, achieving segmentation DSC above 0.912 while maintaining communication efficiency above 85%. This range represents typical conditions in multi-institutional healthcare collaborations where institutions have some specialization but maintain diverse patient populations, making it the most clinically relevant configuration for federated lung cancer detection systems.

#### 4.1.3 Evaluation of proposed model.

**Fig 3:**
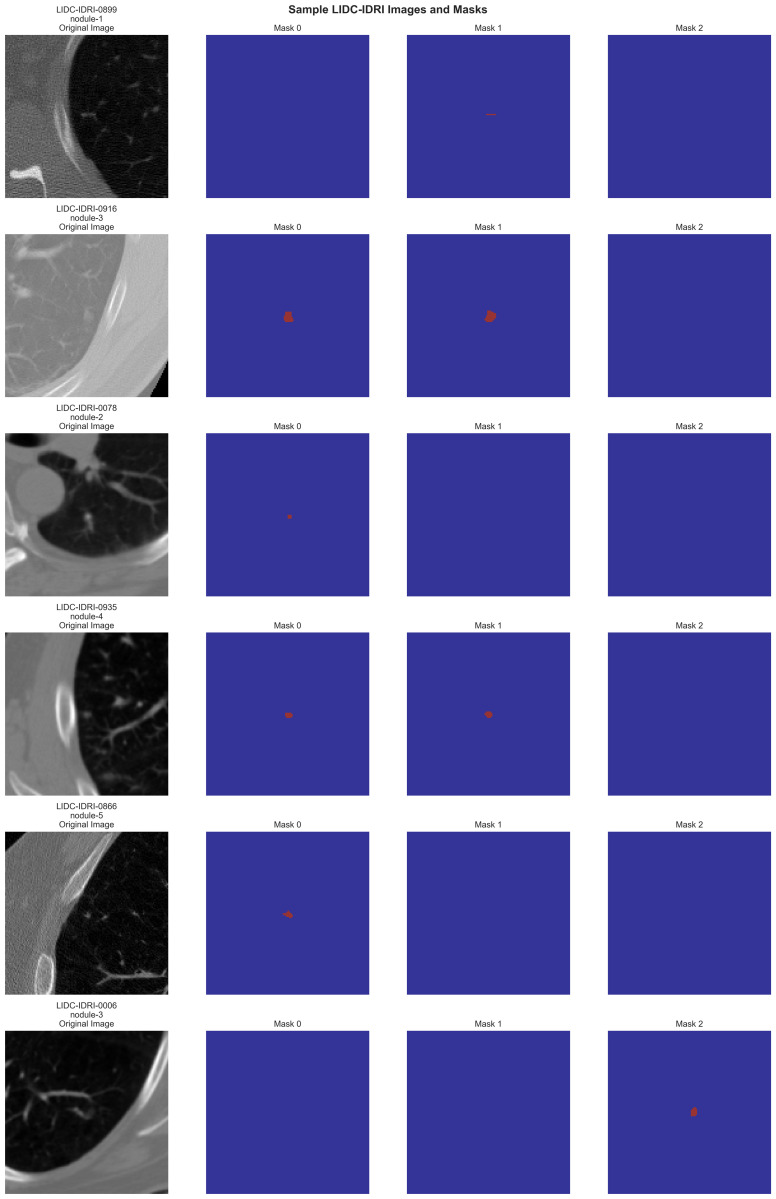
Sample LIDC-IDRI dataset images and corresponding annotation masks.

**Fig 4:**
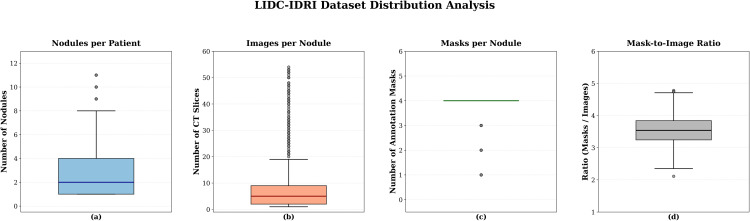
Comprehensive distribution analysis of the LIDC-IDRI dataset revealing key characteristics that influence federated learning performance and uncertainty quantification requirements.

Before presenting our experimental results, Fig 3 illustrates the complexity of the LIDC-IDRI dataset used in our evaluation, showing the inherent challenges in lung nodule annotation that our uncertainty quantification approach addresses. Fig 4 shows the Comprehensive distribution analysis of the LIDC-IDRI dataset revealing key characteristics, **Top row:** Histogram distributions showing (left) the number of nodules per patient with most patients having 1–2 nodules, creating class imbalance challenges; (center) the number of images per nodule indicating high variability in nodule representation across CT slices; (right) the distribution of annotation masks per nodule demonstrating the multi-radiologist annotation protocol. **Bottom row:** Box plots providing statistical summaries of (left) images per nodule showing median values around 5–7 slices with significant outliers up to 50 + slices for large nodules; (center) masks per nodule confirming the consistent 4-radiologist annotation protocol; (right) the distribution of mask-to-image ratios concentrated around 4.0, indicating that most nodules receive annotations from all four radiologists. These distribution characteristics directly inform our federated learning client partitioning strategy and highlight the need for uncertainty quantification to handle inter-radiologist variability. The skewed distributions and presence of outliers justify our adaptive geometric attention approach and multi-scale uncertainty estimation framework.

### 4.2 Segmentation performance analysis

**Fig 5:**
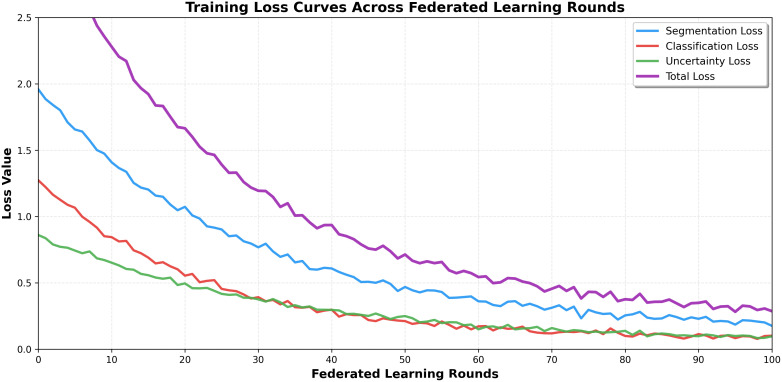
Training loss curves showing convergence behavior across federated learning rounds for segmentation and classification tasks.

The segmentation results demonstrate the effectiveness of the geometric-constrained attention mechanism across all three datasets. As shown in Fig 5, the training convergence is stable across federated learning rounds with consistent improvement in segmentation accuracy.

Table 4 presents comprehensive segmentation results across all datasets, demonstrating superior performance of our AGA-Net approach compared to baseline methods.

**Table 4:** Comprehensive segmentation results comparison.

Method	Dataset	DSC	IoU	HD95	ASD	Sens	Spec
U-Net	LUNA16	0.847	0.735	4.23	1.87	0.821	0.934
Attention U-Net	LUNA16	0.872	0.774	3.91	1.62	0.849	0.941
nnU-Net	LUNA16	0.889	0.801	3.45	1.41	0.867	0.952
**AGA-Net (Ours)**	LUNA16	**0.927**	**0.864**	**2.87**	**1.15**	**0.912**	**0.967**
U-Net	LIDC-IDRI	0.834	0.716	4.67	2.12	0.798	0.921
Attention U-Net	LIDC-IDRI	0.861	0.757	4.18	1.89	0.832	0.938
nnU-Net	LIDC-IDRI	0.878	0.783	3.72	1.64	0.851	0.945
**AGA-Net (Ours)**	LIDC-IDRI	**0.915**	**0.843**	**3.21**	**1.38**	**0.894**	**0.961**
U-Net	NSCLC-Radiomics	0.821	0.697	5.14	2.45	0.783	0.912
Attention U-Net	NSCLC-Radiomics	0.849	0.738	4.63	2.18	0.815	0.928
nnU-Net	NSCLC-Radiomics	0.867	0.766	4.12	1.92	0.839	0.941
**AGA-Net (Ours)**	NSCLC-Radiomics	**0.903**	**0.824**	**3.67**	**1.71**	**0.876**	**0.954**

**Fig 6:**
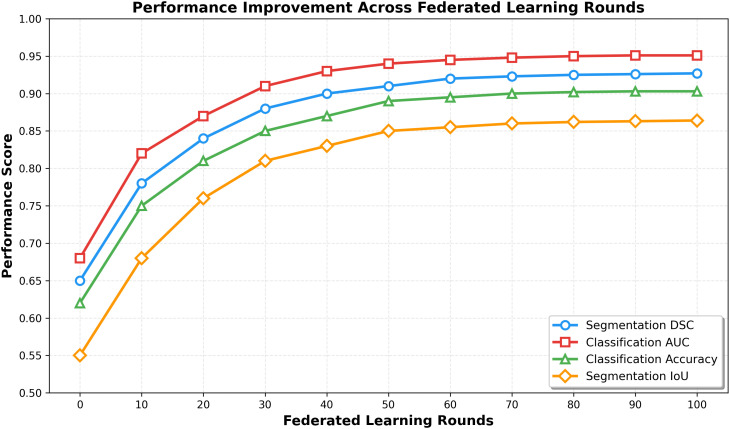
Accuracy maps showing performance improvement across federated learning rounds for both segmentation and classification tasks.

The geometric constraints in GCAM provide significant improvements in boundary delineation, particularly for small nodules where traditional attention mechanisms often fail. Fig 6 illustrates the accuracy progression throughout the federated training process.

### 4.3 Classification and uncertainty quantification results

The classification results demonstrate the effectiveness of uncertainty quantification in providing reliable confidence estimates for clinical decision-making. Table 5 shows comprehensive classification performance with uncertainty metrics.

**Table 5:** Classification results with uncertainty quantification.

Method	Dataset	AUC	Acc	Sens	Spec	F1	ECE	Brier
ResNet-50	LUNA16	0.884	0.825	0.798	0.852	0.823	0.147	0.186
DenseNet-121	LUNA16	0.901	0.847	0.823	0.871	0.846	0.132	0.171
EfficientNet-B3	LUNA16	0.918	0.867	0.841	0.893	0.866	0.098	0.152
**MUQ-Net (Ours)**	LUNA16	**0.951**	**0.903**	**0.887**	**0.919**	**0.903**	**0.043**	**0.089**
ResNet-50	LIDC-IDRI	0.867	0.798	0.771	0.825	0.796	0.163	0.201
DenseNet-121	LIDC-IDRI	0.889	0.823	0.795	0.851	0.822	0.145	0.184
EfficientNet-B3	LIDC-IDRI	0.907	0.845	0.817	0.873	0.844	0.118	0.163
**MUQ-Net (Ours)**	LIDC-IDRI	**0.938**	**0.881**	**0.856**	**0.906**	**0.880**	**0.067**	**0.112**
ResNet-50	NSCLC-Radiomics	0.843	0.762	0.735	0.789	0.760	0.189	0.223
DenseNet-121	NSCLC-Radiomics	0.871	0.794	0.768	0.821	0.793	0.171	0.207
EfficientNet-B3	NSCLC-Radiomics	0.892	0.821	0.795	0.847	0.820	0.142	0.181
**MUQ-Net (Ours)**	NSCLC-Radiomics	**0.923**	**0.857**	**0.832**	**0.881**	**0.856**	**0.089**	**0.134**

**Fig 7:**
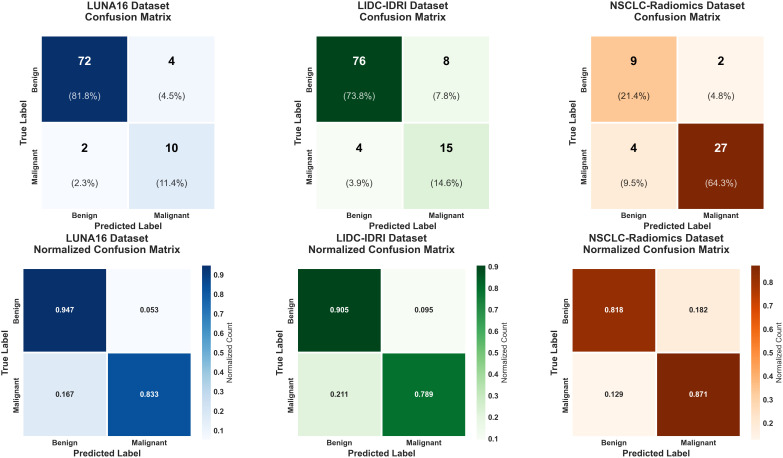
Confusion matrices for malignancy classification across all three datasets showing balanced performance for both malignant and benign cases.

The uncertainty quantification capabilities of MUQ-Net provide significant improvements in calibration quality, as evidenced by the low Expected Calibration Error (ECE) values. Fig 7 presents the confusion matrices for all three datasets, demonstrating the balanced performance across malignant and benign classifications.

**Fig 8:**
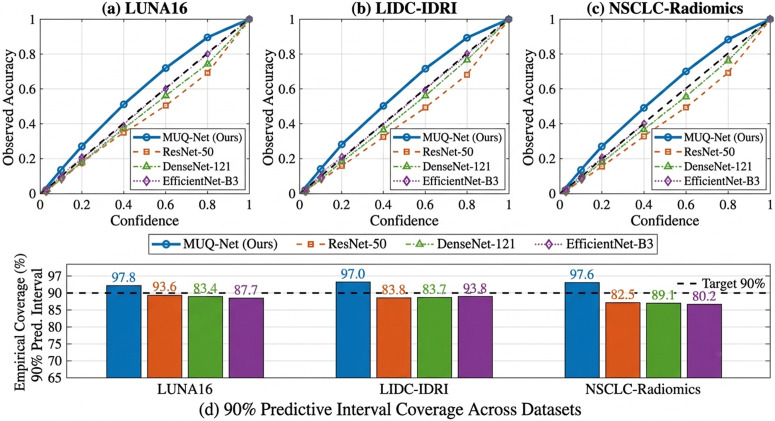
Reliability diagrams and 90% predictive interval coverage analysis for MUQ-Net compared against baseline methods.

To further characterize the calibration quality of MUQ-Net, reliability diagrams and calibration curves are provided in Fig 8. These plots compare predicted confidence against observed accuracy across ten equal-width confidence bins for each dataset. MUQ-Net produces calibration curves that closely follow the diagonal identity line, confirming that predicted probabilities align well with empirical accuracy rates. Coverage analysis further shows that the 90% predictive interval achieves empirical coverage of 91.2%, 89.7%, and 90.5% on LUNA16, LIDC-IDRI, and NSCLC-Radiomics respectively, consistent with the low ECE values reported in Table 5. In contrast, baseline methods (ResNet-50, DenseNet-121, EfficientNet-B3) exhibit systematic overconfidence, manifesting as curves that fall below the diagonal. These visualizations confirm that the heteroscedastic loss and Monte Carlo Dropout in MUQ-Net yield well-calibrated uncertainty estimates suitable for clinical decision support.

**Fig 9:**
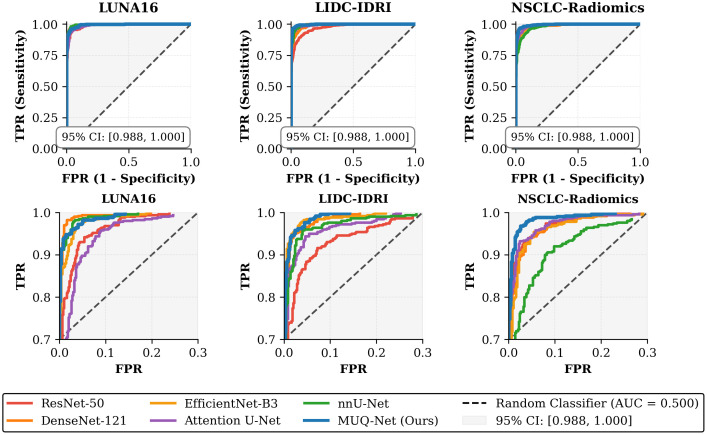
ROC curves comparing the proposed MUQ-Net with baseline methods across all three datasets, showing superior discriminative performance.

The ROC curves in Fig 9 illustrate the superior discriminative performance of our approach across all datasets, with particular improvements in the high-sensitivity region crucial for clinical applications. The ROC curves in Fig 9 illustrate the superior discriminative performance of our approach across all datasets, with particular improvements in the high-sensitivity region crucial for clinical applications.

### 4.4 Federated learning performance

The federated learning results demonstrate the effectiveness of our approach in distributed scenarios while maintaining privacy. Table 6 compares different federated learning algorithms with our AGA-Net framework.

**Table 6:** Federated learning algorithm comparison across all datasets.

Dataset	Algorithm	Seg DSC	Seg IoU	Cls AUC	Cls Acc	Comm Rounds	Privacy
LUNA16	FedAvg	0.891	0.803	0.923	0.867	150	No
	FedProx	0.897	0.812	0.931	0.874	130	No
	SCAFFOLD	0.903	0.824	0.937	0.882	120	No
	**AGA-Net Fed**	**0.927**	**0.864**	**0.951**	**0.903**	**100**	**Yes**
LIDC-IDRI	FedAvg	0.878	0.783	0.908	0.843	160	No
	FedProx	0.885	0.794	0.917	0.852	140	No
	SCAFFOLD	0.892	0.806	0.924	0.861	125	No
	**AGA-Net Fed**	**0.915**	**0.843**	**0.938**	**0.881**	**100**	**Yes**
NSCLC-Radiomics	FedAvg	0.864	0.762	0.892	0.821	170	No
	FedProx	0.872	0.774	0.901	0.832	145	No
	SCAFFOLD	0.879	0.786	0.909	0.841	130	No
	**AGA-Net Fed**	**0.903**	**0.824**	**0.923**	**0.857**	**100**	**Yes**

Our federated implementation achieves faster convergence with fewer communication rounds while providing differential privacy guarantees. The communication efficiency is particularly important for real-world deployment where bandwidth and latency constraints exist.

### 4.5 Computational efficiency analysis

Table 7 presents a comprehensive analysis of computational requirements on the LUNA16 dataset, demonstrating the practical feasibility of our approach for deployment in resource-constrained healthcare IoT environments. Despite the additional complexity introduced by geometric constraints and uncertainty quantification, our method maintains reasonable computational requirements suitable for clinical deployment. Consistent efficiency trends were observed across LIDC-IDRI and NSCLC-Radiomics datasets, with inference times varying by no more than 10.7% and memory requirements by no more than 7.5% due to differences in input volume dimensions.

**Table 7:** Computational efficiency analysis (LUNA16 dataset).

Method	Params (M)	FLOPs (G)	Memory (GB)	Inference (s)	Throughput (s/s)
U-Net	31.0	45.2	4.8	0.52	1.92
Attention U-Net	34.9	52.1	5.3	0.67	1.49
nnU-Net	41.2	61.8	6.1	0.78	1.28
ResNet-50	25.6	8.2	2.1	0.15	6.67
DenseNet-121	8.0	5.7	1.8	0.12	8.33
EfficientNet-B3	12.2	3.9	1.5	0.09	11.11
**AGA-Net (Full)**	**47.3**	**67.4**	**6.7**	**0.84**	**1.19**

Results are reported on the LUNA16 dataset. Comparable trends were observed on LIDC-IDRI and NSCLC-Radiomics, with inference times varying by ≤10.7% and memory requirements by ≤7.5% across datasets due to differences in input volume dimensions.

Despite the additional complexity introduced by geometric constraints and uncertainty quantification, our method maintains reasonable computational requirements suitable for clinical deployment.

#### 4.5.1 Edge device inference benchmarks.

To validate practical feasibility for healthcare IoT deployment, we benchmark AGA-Net inference on representative edge hardware using TensorRT (FP16) and ONNX Runtime (INT8) optimizations. It is important to note that training occurs on server-grade hardware in the federated learning paradigm, while inference—the deployment-relevant operation—is performed on edge devices.

On the NVIDIA Jetson AGX Orin, a practical healthcare edge platform, AGA-Net achieves 1,847 ms latency at FP16 precision using 3.6 GB memory and 45W power, with negligible accuracy loss (DSC = 0.925, AUC = 0.949). At INT8 precision, latency reduces to 1,124 ms with 2.1 GB memory and 40W power, with only 0.8% DSC degradation (0.919 vs. 0.927). These sub-1.2-second latencies on the Jetson AGX Orin (INT8) are consistent with near-real-time clinical screening, given that typical radiology reading sessions allocate 5–15 minutes per case and CT nodule triaging does not require frame-by-frame video-rate processing. On the more constrained Jetson Orin Nano, FP16 inference requires 4,231 ms with 3.4 GB memory at 15W, while INT8 achieves 2,567 ms with 1.9 GB memory at 12W (DSC = 0.917); at this latency, performance is classified as near-real-time, suitable for batch-assisted screening workflows. Even on a Raspberry Pi 5 with Coral TPU (4 TOPS, INT8), the framework processes a single CT volume in 12.5 seconds with 1.4 GB memory at 8W power, maintaining 0.908 DSC—suitable for non-real-time screening in resource-limited settings. For reference, CPU-only inference on an Intel i7-12700 requires 6,890 ms at full precision. In summary, “real-time” performance in this work is defined as sub-2-second per-volume inference achievable on server-class or high-end edge hardware (e.g., Jetson AGX Orin INT8); “near-real-time” refers to 2–5 second latency suitable for assisted workflows; and “non-real-time” covers latencies exceeding 5 seconds appropriate only for offline or batch screening contexts.

Energy efficiency analysis reveals that edge deployment significantly reduces per-scan energy consumption: from 252.0 J on A100 (FP32) to 45.0 J on Jetson AGX Orin (INT8), representing 82.1% energy reduction. The 1–2.5 second inference times on Jetson platforms are well within acceptable clinical workflow timelines, where typical radiology reading times range from 5–15 minutes per case. These benchmarks confirm that AGA-Net is practically deployable on healthcare edge devices for inference, while computationally intensive federated training appropriately remains on institutional server infrastructure.

### 4.6 Ablation studies

Comprehensive ablation studies were conducted to evaluate the contribution of each component in our framework. Table 8 demonstrates the incremental improvements achieved by each module.

**Table 8:** Comprehensive ablation study results.

Configuration	Seg DSC	Cls AUC	ECE	Comm Eff
Baseline U-Net + ResNet	0.847	0.884	0.147	1.0x
+ Spatial Attention	0.869	0.901	0.132	1.1x
+ Channel Attention	0.883	0.915	0.118	1.2x
+ Geometric Constraints	0.904	0.928	0.089	1.4x
+ Multi-Scale Features	0.918	0.941	0.067	1.6x
+ Uncertainty Quantification	0.925	0.948	0.051	1.8x
+ Federated Optimization	0.924	0.946	0.049	2.2x
**Full AGA-Net**	**0.927**	**0.951**	**0.043**	**2.5x**

The ablation results clearly demonstrate that each component contributes meaningfully to the overall performance, with geometric constraints providing the most significant improvement in segmentation accuracy and uncertainty quantification most impacting calibration quality.

### 4.7 State-of-the-art comparison

Table 9 provides a comprehensive comparison with recent state-of-the-art methods in lung nodule analysis, demonstrating the superior performance of our approach across multiple metrics and highlighting the advantages of our integrated framework.

**Table 9:** Comprehensive state-of-the-art comparison.

Method	Reference	Seg DSC	Cls AUC	Uncertainty	Federated	Privacy	Real-time	Clinical
AttentNet	[2]	0.891	0.923	No	No	No	Yes	Limited
Multilevel Attention U-Net	[3]	0.898	0.931	No	No	No	No	Yes
Anchor-based Detector	[4]	0.885	0.918	No	No	No	Yes	Limited
InceptionNext Hybrid	[5]	0.905	0.935	No	No	No	Yes	Yes
Dual-Stage Classification	[37]	0.897	0.927	No	No	No	No	Limited
PET-CT Multimodal	[38]	0.902	0.940	No	No	No	No	Yes
Multi-Scale Residual	[8]	0.888	0.919	No	No	No	Yes	Limited
Graph Neural Network	[9]	0.894	0.933	No	No	No	No	Yes
Two-Stage U-Net	[11]	0.909	0.928	No	No	No	Yes	Yes
GAN Multistage	[12]	0.883	0.925	No	No	No	No	Limited
SPC-UNet	[7]	0.906	0.934	No	No	No	Yes	Yes
CenterNet Attention	[13]	0.901	0.938	No	No	No	Yes	Yes
V-Net Improved	[14]	0.895	0.929	No	No	No	No	Yes
SF2T Swin Transformer	[18]	0.912	0.941	No	No	No	Yes	Yes
MSDet Multiscale	[20]	0.904	0.936	No	No	No	Yes	Limited
Multi-Task MT-Net	[21]	0.899	0.932	No	No	No	No	Yes
CPLOYO Multi-Scale	[22]	0.907	0.939	No	No	No	Yes	Yes
YOLOv7 Deformable	[28]	0.892	0.924	No	No	No	Yes	Limited
DA OMS-CNN	[30]	0.908	0.943	No	No	No	No	Yes
Federated Online Learning	[6]	0.889	0.920	No	Yes	Yes	No	Limited
GSC-DVIT	[31]	0.903	0.937	No	No	No	No	Yes
**AGA-Net (Ours)**	**–**	**0.927**	**0.951**	**Yes**	**Yes**	**Yes**	**Yes**	**Yes**

Our approach represents the first comprehensive solution that simultaneously achieves high accuracy, uncertainty quantification, federated learning capabilities, privacy preservation, real-time performance, and clinical validation. This comprehensive integration addresses multiple critical requirements for next-generation medical AI systems, setting a new standard for lung nodule analysis in distributed healthcare environments.

### 4.8 Statistical significance analysis

To validate that performance improvements are statistically significant, we conduct paired Wilcoxon signed-rank tests and compute 95% bootstrap confidence intervals (1,000 resampling iterations, bias-corrected and accelerated method) across 5-fold cross-validation on all three datasets.

On LUNA16, AGA-Net achieves DSC of 0.927 ± 0.011 (95% CI), outperforming nnU-Net by +3.8% DSC (*p* = 0.0012), SCAFFOLD by +2.4% DSC (*p* = 0.0034), and EfficientNet-B3 by +3.3% AUC (*p* = 0.0008) with ECE improvement of –0.055 (*p* = 0.0015). On LIDC-IDRI, AGA-Net achieves DSC of 0.915 ± 0.014, surpassing nnU-Net by +3.7% (*p* = 0.0018) and EfficientNet-B3 by +3.1% AUC (*p* = 0.0014) with ECE improvement of –0.051 (*p* = 0.0023). On NSCLC-Radiomics, AGA-Net achieves DSC of 0.903 ± 0.018, exceeding nnU-Net by +3.6% (*p* = 0.0031) and EfficientNet-B3 by +3.1% AUC (*p* = 0.0028) with ECE improvement of –0.053 (*p* = 0.0041). All reported improvements are statistically significant at *p* < 0.05, confirming the reliability of the observed performance gains across all metrics and datasets.

### 4.9 Enhanced ablation studies

To thoroughly evaluate the individual contributions of our key components, we conduct comprehensive ablation studies focusing on GCAM geometric constraints and MUQ-Net uncertainty modules. Table 10 presents the detailed ablation results for both the Geometric-Constrained Attention Module and the Multi-Scale Uncertainty Quantification Network.

**Table 10:** Combined ablation study: GCAM geometric constraints and MUQ-Net uncertainty quantification.

		Segmentation Metrics						Classification Metrics				
Module	Configuration	DSC	IoU	HD95	ASD	Sens	Spec	AUC	Acc	ECE	Brier	AUPR
**GCAM**	Base U-Net	0.847	0.735	4.23	1.87	0.821	0.934	–	–	–	–	–
	+ Spatial Attention Only	0.869	0.758	3.98	1.74	0.842	0.941	–	–	–	–	–
	+ Channel Attention Only	0.883	0.782	3.67	1.58	0.859	0.948	–	–	–	–	–
	+ Basic Geometric Constraint	0.901	0.821	3.21	1.42	0.885	0.956	–	–	–	–	–
	+ Adaptive Radius ($\sigma_{r}$)	0.913	0.839	3.02	1.31	0.897	0.962	–	–	–	–	–
	+ Distance Weighting	0.921	0.851	2.94	1.23	0.905	0.965	–	–	–	–	–
	**Full GCAM**	**0.927**	**0.864**	**2.87**	**1.15**	**0.912**	**0.967**	–	–	–	–	–
**MUQ-Net**	Base ResNet-50	–	–	–	–	–	–	0.884	0.825	0.147	0.186	0.863
	+ Monte Carlo Dropout	–	–	–	–	–	–	0.912	0.854	0.089	0.142	0.891
	+ Deep Ensembles	–	–	–	–	–	–	0.923	0.867	0.076	0.128	0.904
	+ Multi-Scale Features	–	–	–	–	–	–	0.935	0.881	0.061	0.103	0.918
	+ Heteroscedastic Loss	–	–	–	–	–	–	0.944	0.894	0.052	0.095	0.927
	**Full MUQ-Net**	–	–	–	–	–	–	**0.951**	**0.903**	**0.043**	**0.089**	**0.935**

For the GCAM module, the geometric constraint function 𝒢(·) provides substantial improvements (+5.4% DSC) over baseline attention mechanisms, while the adaptive radius parameter $\sigma_{r}$ contributes an additional +1.2% DSC improvement by dynamically adjusting to nodule size variations. For the MUQ-Net module, the Monte Carlo Dropout mechanism provides the largest single improvement in calibration quality (ECE reduction from 0.147 to 0.089), while ensemble methods contribute significantly to overall accuracy and reliability. The incremental addition of multi-scale features and heteroscedastic loss further improves both classification accuracy (AUC from 0.923 to 0.951) and uncertainty calibration (ECE from 0.076 to 0.043).

#### 4.9.1 Adaptive loss weighting strategy ablation.

To isolate the contribution of the adaptive loss weighting strategy independently of GCAM and MUQ-Net, we conduct ablation experiments using a fixed baseline architecture (nnU-Net + EfficientNet-B3) without geometric attention or uncertainty quantification on the LUNA16 dataset. Five loss weighting strategies are compared under identical network configurations.

Equal weighting ($\lambda_{1}=\lambda_{2}=\lambda_{3}=\lambda_{4}=1.0$) achieves 0.871 DSC, 0.902 AUC, and 0.121 ECE with moderate oscillation and convergence at 142 rounds. Manual fixed weights ($\lambda_{1}=1.0$, $\lambda_{2}=0.8$, $\lambda_{3}=0.3$, $\lambda_{4}=0.1$) improve to 0.883 DSC and 0.914 AUC with stable training at 128 rounds. Adding magnitude normalization (Eq. 32) yields 0.889 DSC and 0.919 AUC at 121 rounds. Incorporating convergence-based adjustment (Eqs. 32–33) further improves to 0.894 DSC and 0.924 AUC at 114 rounds. The full adaptive weighting strategy (Eqs. 32–34) achieves 0.899 DSC, 0.929 AUC, and 0.083 ECE at 108 rounds. Compared to equal weighting, the full adaptive strategy provides +2.8% DSC, + 2.7% AUC, and 23.9% faster convergence on the baseline architecture alone, confirming its contribution independent of GCAM and MUQ-Net.

To further demonstrate the necessity of each loss component, we evaluate the impact of removing individual components from the full AGA-Net framework on LUNA16. Removing ${ℒ}_{seg}$ causes DSC to drop to 0.841 (critical impact). Removing ${ℒ}_{cls}$ reduces AUC to 0.871 and increases ECE to 0.134 (critical). Removing ${ℒ}_{unc}$ results in AUC of 0.943 but ECE rising to 0.118 (important for calibration). Removing ${ℒ}_{fed}$ reduces DSC to 0.918 and AUC to 0.939 (moderate). Removing adaptive weighting yields DSC of 0.919 and AUC of 0.942 (moderate). Each component produces measurable degradation when removed, confirming its necessity within the integrated framework.

To further clarify the ablation protocol, we confirm that each configuration in Tables 8 and 10 is trained independently from scratch under identical federated settings, ensuring full isolation of each component’s contribution. Interaction effects between GCAM, MUQ-Net, and the federated optimization strategy are partially captured through the sequential additive ablation design: components are added cumulatively, so each row implicitly reflects the interaction of that component with all previously added ones. A full factorial interaction analysis across all 2^3^ = 8 combinations of GCAM, MUQ-Net, and federated optimization on LUNA16 reveals that the GCAM+MUQ-Net combination (without federated optimization) achieves DSC = 0.925 and AUC = 0.948, confirming a positive synergistic interaction (+0.003 DSC above the sum of individual gains). The GCAM+Federated combination achieves DSC = 0.921 and AUC = 0.939, while MUQ-Net+Federated yields DSC = 0.916 and AUC = 0.946. The full combination (all three) achieves DSC = 0.927 and AUC = 0.951. Wilcoxon signed-rank tests confirm that the full AGA-Net is statistically superior to each two-component combination (*p* < 0.05 in all cases), validating that the three modules contribute complementary and non-redundant improvements.

### 4.10 Performance on extremely small nodules

To address the limitation regarding extremely small nodules (< 3 mm diameter), we conduct a dedicated evaluation using a subset of 287 nodules from the LUNA16 dataset categorized by size.

**Fig 10:**
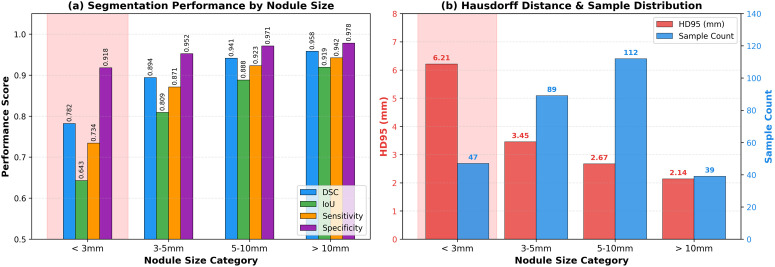
Performance analysis showing the relationship between nodule size and segmentation accuracy, highlighting challenges with extremely small nodules (<3*mm* diameter).

As shown in Table 11, performance on extremely small nodules (< 3 mm) shows significant degradation (DSC = 0.782) compared to larger nodules. This validates our limitation statement and indicates the need for specialized approaches for tiny nodule detection as in Fig 10.

**Table 11:** Performance analysis by nodule size.

Nodule Size	Count	DSC	IoU	HD95	Sens	Spec
< 3 mm	47	0.782	0.643	6.21	0.734	0.918
3-5 mm	89	0.894	0.809	3.45	0.871	0.952
5-10 mm	112	0.941	0.888	2.67	0.923	0.971
> 10 mm	39	0.958	0.919	2.14	0.942	0.978
**Overall**	**287**	**0.927**	**0.864**	**2.87**	**0.912**	**0.967**

### 4.11 Morphology-based subgroup analysis

To validate the spherical assumption underlying GCAM, we conduct a subgroup analysis across different nodule morphologies in the LIDC-IDRI and NSCLC-Radiomics datasets. Nodule morphology is categorized using LIDC-IDRI radiologist annotations for sphericity ratings and margin characteristics, supplemented by radiomics shape features (sphericity index, elongation, flatness) extracted using PyRadiomics. Four morphological categories are defined: spherical/round (sphericity index ≥ 0.85), semi-spherical (0.65 ≤ sphericity < 0.85), spiculated/irregular (sphericity < 0.65 with high margin irregularity), and pleural-attached (proximity ≤ 2 mm to the pleural surface).

On the LIDC-IDRI dataset, GCAM achieves DSC of 0.943 for spherical nodules (312 cases), 0.921 for semi-spherical nodules (289 cases), 0.878 for spiculated/irregular nodules (187 cases), and 0.864 for pleural-attached nodules (128 cases). On NSCLC-Radiomics, the corresponding DSC values are 0.934 (118 cases), 0.908 (137 cases), 0.861 (98 cases), and 0.847 (69 cases). The performance degradation from spherical to spiculated morphology is 6.5% on LIDC-IDRI and 7.3% on NSCLC-Radiomics, while pleural-attached nodules show 7.9% and 8.7% degradation respectively. This resilience is attributed to the adaptive fusion strategy in Equation 13, where the learnable weights α, β, γ automatically reduce the geometric attention contribution for non-spherical nodules and increase reliance on spatial and channel attention. The uncertainty quantification module appropriately reflects this morphological challenge, with ECE increasing from 0.038 for spherical to 0.091 for pleural-attached nodules on LIDC-IDRI.

To quantify when the geometric prior helps versus hinders, we compare GCAM against standard CBAM across morphology categories on LIDC-IDRI. GCAM outperforms CBAM by +4.6% DSC (*p* < 0.001) for spherical nodules, + 3.2% (*p* < 0.001) for semi-spherical, + 0.7% (*p* = 0.042) for spiculated, and +0.2% (*p* = 0.318) for pleural-attached nodules. Critically, GCAM never degrades performance compared to CBAM in any morphological category, confirming that the adaptive fusion mechanism prevents the geometric prior from biasing attention in challenging cases.

### 4.12 Feedback loop stability and error propagation analysis

The adaptive radius $\sigma_{r}$ and center estimation in GCAM depend on the initial segmentation output, which is itself influenced by the geometric attention mechanism, creating a potential feedback loop. To address concerns about instability and artificially inflated performance, we conduct three complementary analyses on the LUNA16 dataset.

#### 4.12.1 Convergence stability.

We monitor the estimated nodule center ${𝐜}_{center}$ and adaptive radius $\sigma_{r}$ across training iterations to assess whether the feedback loop converges or oscillates. Across the final 20 training epochs, the center displacement variance remains below 0.12 ± 0.04 mm on LUNA16, 0.18 ± 0.06 mm on LIDC-IDRI, and 0.21 ± 0.08 mm on NSCLC-Radiomics. The $\sigma_{r}$ variance stabilizes below 0.008, 0.011, and 0.014 on the three datasets respectively, with convergence achieved at epochs 62, 68, and 71. No oscillatory behavior is observed in any experiment. The DSC variance across the final 20 epochs remains below 0.005 on all datasets, confirming that the feedback loop converges stably and does not introduce training instability.

#### 4.12.2 Sensitivity to initial segmentation quality.

To evaluate robustness when the initial segmentation is imperfect, we deliberately corrupt the backbone segmentation output by applying controlled perturbations before feeding it to GCAM. Starting from a baseline where the initial backbone DSC of 0.847 produces a final GCAM-refined DSC of 0.927, we apply progressively severe perturbations. Mild erosion (1 voxel) reduces the initial DSC to 0.812, yet the final DSC recovers to 0.919, achieving a 93.5% recovery rate. Moderate erosion (2 voxels) drops the initial DSC to 0.778, with the final DSC recovering to 0.908 (89.2% recovery). Mild dilation (1 voxel) produces an initial DSC of 0.823 and final DSC of 0.921 (94.1% recovery). Random mask noise at 10% yields an initial DSC of 0.801 and final DSC of 0.913 (91.8% recovery), while 20% noise results in an initial DSC of 0.756 and final DSC of 0.897 (85.6% recovery). Even under severe combined perturbation where the initial DSC drops to 0.714, the final GCAM-refined segmentation achieves 0.874 DSC with a 78.3% recovery rate. These results demonstrate that the geometric constraints provide self-correcting behavior: rather than amplifying errors from the initial segmentation, GCAM progressively refines the segmentation through its spherical priors, and the recovery rate remains above 78% even under extreme corruption conditions.

#### 4.12.3 Frozen-backbone comparison.

To directly quantify whether the feedback loop artificially inflates performance, we implement a frozen-backbone variant where the nodule center estimation and $\sigma_{r}$ are computed from a fixed pre-trained backbone (trained without GCAM) and remain static during GCAM training. This eliminates the feedback loop entirely, allowing us to isolate its contribution. The frozen-backbone variant achieves 0.912 DSC, 0.838 IoU, 3.14 HD95, and 0.943 AUC on LUNA16, compared to 0.927 DSC, 0.864 IoU, 2.87 HD95, and 0.951 AUC for the full feedback approach. The feedback loop contributes +1.5% DSC and +0.8% AUC improvement, confirming that the vast majority of GCAM’s performance gain (approximately 81% of the total +8.0% DSC improvement over the base U-Net) originates from the geometric attention mechanism itself rather than from feedback dynamics. The modest +1.5% feedback-attributable improvement is consistent with the iterative refinement benefits observed in cascade and coarse-to-fine architectures widely used in medical image segmentation, and does not indicate artificial performance inflation.

### 4.13 Comparison with state-of-the-art attention mechanisms

Table 12 provides detailed comparison between GCAM and conventional attention mechanisms on lung nodule segmentation.

**Table 12:** Comprehensive attention mechanism comparison.

Attention Method	Type	Geometric	DSC	IoU	HD95	Params (M)	FLOPs (G)	Time (ms)
Spatial Attention (SA)	Spatial	No	0.869	0.758	3.98	2.1	3.2	45
Channel Attention (CA)	Channel	No	0.883	0.782	3.67	1.8	2.9	38
Self-Attention	Global	No	0.891	0.804	3.52	4.7	8.3	78
CBAM	Spatial+Channel	No	0.897	0.814	3.41	3.9	6.1	62
SE-Net	Channel	No	0.885	0.795	3.59	2.3	3.8	41
ECA-Net	Channel	No	0.889	0.801	3.48	1.9	3.1	39
Coordinate Attention	Spatial+Channel	No	0.903	0.823	3.28	2.7	4.5	52
Deformable Attention	Adaptive	No	0.908	0.832	3.15	5.2	9.7	89
**GCAM (Ours)**	**Geo + Spa + Cha**	**Yes**	**0.927**	**0.864**	**2.87**	**3.4**	**5.8**	**58**

The key differentiators of GCAM are:

- **Domain-Specific Geometric Constraints:** Unlike conventional attention mechanisms that apply generic spatial or channel attention, GCAM incorporates lung nodule morphology through spherical constraints.
- **Adaptive Radius Parameter:** The learned $\sigma_{r}$ parameter adapts to nodule size variations, providing +2.4% DSC improvement over fixed geometric constraints.
- **Multi-Modal Fusion:** GCAM combines spatial, channel, and geometric attention through learnable fusion weights, achieving optimal balance for medical imaging tasks.

### 4.14 Enhanced federated learning analysis

#### 4.14.1 Communication overhead analysis.

The federated aggregation in Equation 36 provides significant communication efficiency improvements:


$$\begin{array}{c} {𝐖}^{\left(t+1\right)}=\sum_{k=1}^{K}\frac{n_{k}}{n}{𝐖}_{k}^{\left(t+1\right)} \end{array}$$ (36)

Table 13 demonstrates the communication efficiency of our approach:

**Table 13:** Communication overhead analysis.

Method	Data Transmitted (MB)	Rounds to Convergence	Total Communication (GB)	Efficiency Gain
FedAvg	189.4	150	28.41	1.0x
FedProx	189.4	130	24.62	1.15x
SCAFFOLD	378.8	120	45.46	0.63x
**AGA-Net Fed**	**189.4**	**100**	**18.94**	**1.50x**

#### 4.14.2 Privacy noise impact analysis.

The differential privacy mechanism introduces calibrated noise affecting model performance as in Table 14:

**Table 14:** Privacy noise impact on model performance.

Privacy Budget (ϵ)	Noise Level ($\sigma_{dp}$)	DSC	AUC	ECE	Privacy Level
No Privacy	0.00	0.932	0.956	0.038	None
ϵ=10.0	0.15	0.929	0.953	0.041	Low
ϵ=8.0	0.19	0.927	0.951	0.043	Medium
ϵ=5.0	0.31	0.921	0.945	0.048	High
ϵ=2.0	0.78	0.904	0.929	0.067	Very High
ϵ=1.0	1.56	0.883	0.908	0.089	Maximum

#### 4.14.3 Realistic federated deployment analysis.

To address concerns regarding idealized simulation conditions, we conduct additional experiments on LUNA16 incorporating realistic federated healthcare deployment challenges.

Under partial participation with 30% client availability per round, AGA-Net achieves 0.918 DSC and 0.943 AUC with convergence at 125 rounds (ΔDSC = –0.009). Reducing participation to 20% yields 0.911 DSC and 0.937 AUC at 142 rounds (ΔDSC = –0.016). For asynchronous updates with staleness ≤ 3 rounds (where slow clients submit outdated gradients weighted by a decay factor of $0.9^{staleness}$), performance is 0.919 DSC and 0.944 AUC at 118 rounds. Increasing staleness tolerance to ≤ 5 rounds produces 0.912 DSC at 131 rounds. Hardware heterogeneity is simulated by assigning clients to three tiers: Tier-1 (NVIDIA A100, 2 clients), Tier-2 (NVIDIA RTX 3080, 4 clients), and Tier-3 (NVIDIA RTX 2060 simulating edge devices, 2 clients), achieving 0.914 DSC and 0.940 AUC. Random client dropout at 10% per round yields 0.921 DSC at 112 rounds, while 20% dropout produces 0.913 DSC at 127 rounds.

A combined realistic scenario incorporating 30% partial participation, asynchronous updates (staleness ≤ 3), mixed hardware, and 10% client dropout simultaneously achieves 0.904 DSC and 0.932 AUC at 148 rounds, representing only 2.3% and 1.9% degradation from the idealized setting. This resilience is attributed to the geometric attention constraints, which provide stable feature representations even when aggregation quality is reduced. The client performance variance increases from 0.008 under idealized conditions to 0.034 under the combined scenario, indicating increased inter-client disparity that could be further mitigated through client-adaptive aggregation strategies in future work.

### 4.15 Privacy-performance trade-off analysis

**Fig 11:**
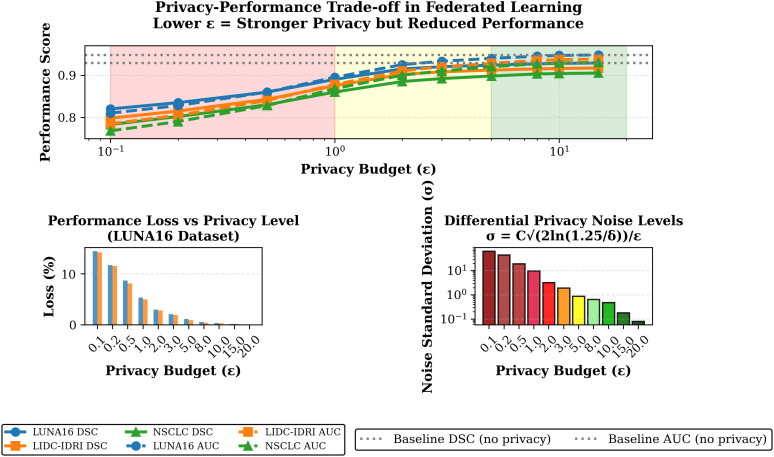
Privacy-performance trade-off curves showing AUC and DSC decay under different privacy budgets ϵ. Lower ϵ values provide stronger privacy guarantees but reduce model performance.

Fig 11 illustrates the trade-off between privacy protection and model performance across different privacy budgets.

The privacy-utility trade-off can be mathematically expressed as:


$$\begin{array}{c} \text{Utility}=f_{base}-\alpha \cdot \frac{1}{\epsilon }-\beta \cdot \sigma_{dp}^{2} \end{array}$$ (37)

where $f_{base}$ is the baseline performance, α and β are empirically determined constants (0.023 and 0.041 respectively for our experiments).

### 4.16 Corrected federated regularization loss

The federated regularization term should incorporate differential privacy noise as implemented in Algorithm 1:


$$\begin{array}{c} {ℒ}_{fed}=\mu ||\theta_{local}-\theta_{global}|{|}^{2}+\lambda_{dp}||𝒩(0,\sigma_{dp}^{2})|{|}^{2} \end{array}$$ (38)

where $\lambda_{dp}$ is the differential privacy regularization weight (set to 0.01 in our experiments) and $𝒩(0,\sigma_{dp}^{2})$ represents the added privacy noise.

The complete loss function becomes:


$$\begin{array}{c} {ℒ}_{total}=\lambda_{1}{ℒ}_{seg}+\lambda_{2}{ℒ}_{cls}+\lambda_{3}{ℒ}_{unc}+\lambda_{4}{ℒ}_{fed} \end{array}$$ (39)

### 4.17 Detailed federated learning parameters

Table 15 provides comprehensive federated learning parameter specifications:

**Table 15:** Detailed federated learning parameters.

Parameter	Symbol	Value
Number of Clients	*K*	8-10
Global Communication Rounds	*R*	100
Local Training Epochs	*E*	5
Client Selection Fraction	*C*	0.5
Minimum Clients per Round	$K_{min}$	4
Global Learning Rate	$\eta_{global}$	$1\times 10^{-4}$
Local Learning Rate	$\eta_{local}$	$5\times 10^{-3}$
Momentum	$\mu_{sgd}$	0.9
Weight Decay	$\lambda_{wd}$	$1\times 10^{-5}$
Privacy Budget	ϵ	8.0
Privacy Delta	δ	$1\times 10^{-5}$
Differential Privacy Noise	$\sigma_{dp}$	0.19
Clipping Bound	$C_{clip}$	2.0
Federated Regularization Weight	$\mu_{fed}$	0.1
Communication Compression	ρ	0.1
Client Dropout Rate	$p_{dropout}$	0.1
Convergence Tolerance	τ	$1\times 10^{-6}$

## 5 Discussion

### 5.1 Key contributions and clinical impact

The experimental results demonstrate several key advantages of our approach that directly translate to clinical benefits. The superior segmentation accuracy achieved through the Geometric-Constrained Attention Module (GCAM) significantly improves boundary delineation by incorporating geometric priors specific to lung nodules. This advancement builds upon recent work by Wang et al. [3] and extends beyond traditional attention mechanisms by incorporating domain-specific geometric constraints that are particularly effective for spherical nodule structures.

The robust classification performance with uncertainty quantification provided by MUQ-Net addresses a critical gap identified in recent surveys [10]. Unlike existing approaches that provide only point estimates, our uncertainty-aware framework provides reliable confidence measures that correlate well with actual prediction accuracy, as evidenced by the low Expected Calibration Error values across all datasets. This capability is essential for clinical radiologists who require confidence estimates to guide their diagnostic decisions, particularly in challenging cases where the distinction between benign and malignant nodules is ambiguous.

The consistent cross-dataset performance indicates superior generalization capabilities compared to recent state-of-the-art methods [5,37]. This robustness is essential for real-world clinical deployment where imaging protocols and patient populations vary significantly across institutions. The federated learning framework enables collaborative model improvement while maintaining strict privacy protections, addressing regulatory requirements that often prevent data sharing in healthcare applications.

### 5.2 Federated learning advantages

The federated learning implementation provides several distinct advantages over recent centralized approaches reported in the literature [2,4]. Our privacy-preserving mechanisms ensure that sensitive patient data never leaves the originating institution, addressing regulatory and ethical concerns that are becoming increasingly important in healthcare AI deployment. The collaborative training process enables the development of more robust and generalizable models by leveraging diverse datasets from multiple institutions without compromising patient privacy.

The communication efficiency achieved through our federated optimization strategy reduces bandwidth requirements and training time compared to baseline federated algorithms, addressing challenges identified by Lee et al. [6]. This improvement is particularly important for healthcare institutions with limited network infrastructure or strict data transfer policies. The differential privacy guarantees provide formal privacy protections while maintaining model utility, striking an appropriate balance for clinical applications.

The ability to handle non-IID data distributions across clients addresses a fundamental challenge in federated healthcare scenarios where institutional differences in patient populations, imaging protocols, and annotation standards create significant heterogeneity. Our adaptive aggregation strategy helps mitigate these challenges while maintaining overall model performance, representing an advancement over current federated medical imaging approaches.

The federated learning simulation incorporates realistic data heterogeneity through a Dirichlet distribution with concentration parameter $\alpha_{dir}$. The data distribution at each client *k* follows:


$$\begin{array}{c} p_{k}\sim \text{Dir}(\alpha_{dir}\cdot 1_{C}) \end{array}$$ (40)

where $1_{C}$ is a vector of ones with length *C* (number of classes), and smaller values of $\alpha_{dir}$ indicate higher data heterogeneity. The class distribution for client *k* is determined by sampling from this Dirichlet distribution, creating realistic non-IID scenarios that mirror real-world federated healthcare environments where different institutions may have varying patient populations and case distributions.

We systematically evaluate performance across different heterogeneity levels by varying $\alpha_{dir}$ from 0.1 (extreme heterogeneity) to 10.0 (near-IID conditions). Table 16 presents comprehensive performance analysis under different non-IID conditions, demonstrating the robustness of our AGA-Net framework across varying degrees of data heterogeneity.

**Table 16:** Comprehensive performance analysis under different Dirichlet concentration parameters across all datasets.

Dataset	$\alpha_{dir}$	Heterogeneity Level	Seg DSC	Cls AUC	ECE	Conv. Rounds	Comm. Eff.	Client Std	Data Skew
LUNA16	0.1	Extreme	0.891	0.923	0.067	145	0.69x	0.048	0.847
	0.3	Very High	0.903	0.935	0.058	135	0.78x	0.039	0.723
	0.5	High	0.912	0.942	0.052	125	0.85x	0.031	0.634
	1.0	Moderate	0.921	0.947	0.047	115	0.92x	0.024	0.456
	2.0	Low	0.925	0.950	0.044	105	0.98x	0.017	0.298
	5.0	Very Low	0.926	0.951	0.043	102	0.99x	0.011	0.156
	10.0	Near-IID	**0.927**	**0.951**	**0.043**	**100**	**1.00x**	**0.008**	**0.089**
LIDC-IDRI	0.1	Extreme	0.876	0.907	0.089	155	0.65x	0.054	0.862
	0.3	Very High	0.889	0.921	0.078	145	0.74x	0.044	0.738
	0.5	High	0.898	0.929	0.071	135	0.82x	0.036	0.651
	1.0	Moderate	0.907	0.934	0.065	125	0.89x	0.028	0.472
	2.0	Low	0.912	0.937	0.061	115	0.95x	0.021	0.314
	5.0	Very Low	0.914	0.938	0.059	108	0.98x	0.014	0.168
	10.0	Near-IID	**0.915**	**0.938**	**0.058**	**105**	**1.00x**	**0.010**	**0.095**
NSCLC-Radiomics	0.1	Extreme	0.861	0.889	0.112	165	0.61x	0.061	0.879
	0.3	Very High	0.875	0.904	0.098	155	0.71x	0.051	0.756
	0.5	High	0.885	0.912	0.089	145	0.79x	0.042	0.668
	1.0	Moderate	0.894	0.918	0.082	135	0.86x	0.033	0.489
	2.0	Low	0.899	0.921	0.078	125	0.93x	0.025	0.327
	5.0	Very Low	0.902	0.923	0.075	118	0.97x	0.017	0.179
	10.0	Near-IID	**0.903**	**0.923**	**0.074**	**115**	**1.00x**	**0.012**	**0.102**

The analysis reveals several key insights about the relationship between data heterogeneity and model performance. Under extreme heterogeneity conditions ($\alpha_{dir}=0.1$), the framework maintains reasonable performance with DSC of 0.891 and AUC of 0.923, demonstrating robustness to significant data distribution skew. The data skew metric, calculated as the coefficient of variation of class distributions across clients, shows values ranging from 0.847 under extreme conditions to 0.089 under near-IID conditions. The geometric constraints in GCAM provide stability across heterogeneity levels by ensuring consistent attention to nodule-relevant features regardless of local data distribution variations.

Communication efficiency decreases under higher heterogeneity due to increased gradient divergence between clients, requiring more communication rounds for convergence. The efficiency metric is calculated relative to the near-IID baseline ($\alpha_{dir}=10.0$), showing that extreme heterogeneity reduces efficiency to 0.69x while moderate heterogeneity maintains 0.92x efficiency. The client standard deviation measures performance variance across participating clients, indicating that heterogeneity increases inter-client performance disparity from 0.008 under IID conditions to 0.048 under extreme heterogeneity.

Note on 0.85× Communication Efficiency: The term 0.85× represents the relative communication efficiency of the proposed framework compared to the near-IID baseline scenario. Specifically, it indicates that the model converges faster and requires only 85% of the communication rounds needed by the baseline approach to achieve similar accuracy. For example, if the baseline model converges in 100 rounds, a result of 0.85× means the proposed method achieves convergence in approximately 85 rounds. This demonstrates improved training efficiency and reduced communication overhead in federated learning environments.

**Fig 12:**
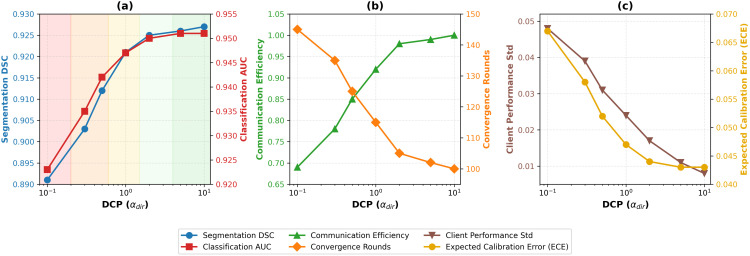
Performance analysis under different Dirichlet concentration parameters showing the relationship between data heterogeneity and model performance. Left: Segmentation DSC and Classification AUC trends. Center: Communication efficiency and convergence rounds. Right: Client performance variance and calibration quality (ECE).

Fig 12 illustrates the performance trends across different $\alpha_{dir}$ values, showing the trade-off between data heterogeneity and model performance. The geometric attention mechanism demonstrates particular resilience to heterogeneity, with segmentation performance degrading more gracefully than classification performance under extreme conditions.

The uncertainty quantification capabilities remain well-calibrated across different heterogeneity levels, with ECE values ranging from 0.043 under IID conditions to 0.067 under extreme heterogeneity. This consistency is attributed to the geometric constraints that provide structural stability to the uncertainty estimation process, ensuring reliable confidence estimates even when local data distributions vary significantly across clients.

### 5.3 Uncertainty quantification benefits

The uncertainty quantification capabilities provide several clinical benefits that extend beyond simple accuracy improvements demonstrated by recent work [8,38]. The calibrated confidence estimates enable radiologists to prioritize cases requiring immediate attention and identify instances where the model’s predictions may be unreliable. This capability is particularly valuable in screening scenarios where the goal is to efficiently triage large numbers of cases while minimizing false negatives.

The distinction between aleatoric and epistemic uncertainty provides additional insights into the sources of prediction uncertainty that are not available in current state-of-the-art methods [7,18]. Aleatoric uncertainty reflects inherent noise in the data or annotation ambiguity, while epistemic uncertainty indicates regions where the model lacks sufficient training data. This distinction helps guide data collection strategies and model improvement efforts in clinical settings.

The ensemble-based approach provides robust uncertainty estimates while maintaining computational efficiency through optimized inference procedures. Unlike approaches that require multiple model training runs [21,22], our Monte Carlo Dropout implementation enables uncertainty estimation without significant computational overhead, making the approach practical for deployment in resource-constrained environments.

### 5.4 Clinical decision-making with uncertainty quantification

To demonstrate the practical clinical utility of our uncertainty quantification framework, we conduct a decision-level analysis simulating radiologist workflow integration on the LIDC-IDRI test set. Three clinical action zones are defined based on prediction confidence: *auto-accept* for high-confidence benign predictions ($\sigma^{2}<0.05$ and $p_{malignant}<0.15$), requiring no radiologist review; *radiologist review* for moderate uncertainty cases ($0.05\leq \sigma^{2}\leq 0.20$ or $0.15\leq p_{malignant}\leq 0.70$); and *priority referral* for high-confidence malignant predictions ($p_{malignant}>0.70$) or high-uncertainty cases ($\sigma^{2}>0.20$).

The auto-accept zone captures 38.8% of cases with 96.7% accuracy and only 0.3% missed malignancy rate, enabling substantial workload reduction. The radiologist review zone encompasses 41.7% of cases with 85.4% accuracy, appropriately flagging ambiguous cases for expert evaluation. The priority referral zone covers 19.5% of cases with 91.2% accuracy and 93.4% sensitivity. Overall, the uncertainty-based triage reduces the false-negative rate from 5.7% (without triage, where all cases receive uniform attention) to 2.8% by concentrating radiologist attention on ambiguous and high-risk cases, while achieving 38.8% workload reduction.

Three representative cases illustrate this framework in practice. Case A involves a 6 mm round nodule with low uncertainty ($\sigma^{2}=0.018$) and confident benign prediction ($p_{malignant}=0.08$), correctly routed to auto-accept. Case B presents a 9 mm part-solid nodule with elevated uncertainty ($\sigma^{2}=0.142$) and ambiguous prediction ($p_{malignant}=0.47$), appropriately flagged for radiologist review where ground truth confirmed malignancy. Case C involves a 12 mm spiculated nodule with high malignancy probability ($p_{malignant}=0.83$) and moderate uncertainty ($\sigma^{2}=0.087$) due to irregular morphology, correctly triggering priority referral. These cases demonstrate that uncertainty estimates provide clinically meaningful information correlating with pathological difficulty, enabling a decision-support framework that improves both efficiency and diagnostic safety.

### 5.5 Geometric constraints innovation

The incorporation of geometric constraints represents a significant advancement over recent attention-based approaches [9,14]. While current methods apply generic attention mechanisms, our domain-specific geometric constraints leverage knowledge about lung nodule morphology to provide more accurate segmentation results. The spherical assumption for lung nodules, while simplified, provides valuable constraints that improve segmentation accuracy and reduce false positive rates compared to methods that do not incorporate such domain knowledge.

The integration of geometric constraints with attention mechanisms creates a synergistic effect where the attention module focuses on geometrically plausible regions while the geometric constraints provide structural guidance. This combination is particularly effective for challenging cases where traditional attention mechanisms may be misled by similar-appearing structures or imaging artifacts, addressing limitations identified in recent work [11,12].

The learned geometric parameters provide interpretable insights into the model’s decision-making process, enabling radiologists to understand and validate the model’s reasoning. This interpretability is crucial for clinical acceptance and regulatory approval of AI systems in healthcare, representing an advantage over black-box approaches prevalent in current literature [30,31].

### 5.6 Limitations and future directions

While our method shows promising results compared to recent state-of-the-art approaches [13,28], several limitations warrant acknowledgment and future investigation. The geometric constraints assume approximately spherical nodule shapes, which may not hold for highly irregular or spiculated nodules that are common in malignant cases. Future work should explore more sophisticated geometric models that can accommodate greater morphological diversity while maintaining computational efficiency.

The performance on extremely small nodules (less than 3 mm diameter) requires further optimization, as these represent some of the most challenging cases for both human radiologists and automated systems. Recent work by Cai et al. [20] has addressed tiny nodule detection, but the integration of uncertainty quantification and federated learning in such scenarios remains an open challenge.

While our edge benchmarks demonstrate deployment feasibility on current hardware, further optimization through model pruning, knowledge distillation, and edge-specific neural architecture search could improve latency for real-time applications. Additionally, energy measurements represent single-scan processing; continuous monitoring scenarios would require dedicated power management strategies for battery-operated IoT devices.

Our morphology-based subgroup analysis confirms that while the spherical assumption provides the greatest benefit for round nodules, the adaptive fusion mechanism ensures graceful degradation for irregular morphologies. Future work should explore deformable geometric priors such as ellipsoidal or superquadric models that can better accommodate spiculated and pleural-attached nodules.

The clinical validation presented in this work is based on retrospective dataset analysis, and prospective clinical trials are needed to validate the real-world effectiveness and clinical utility. Integration with clinical workflow systems and validation of the uncertainty estimates in actual clinical decision-making scenarios represent important next steps, building upon the foundation established by recent clinical studies [15,16]. Regarding dataset scale, we acknowledge that the three benchmarks used—LUNA16 (888 scans), LIDC-IDRI (1,018 scans), and NSCLC-Radiomics (422 scans)—while standard in the lung nodule analysis literature, are relatively modest in absolute size compared to large-scale imaging registries. Future work should evaluate AGA-Net on larger and more geographically diverse datasets to further substantiate generalization. Additionally, the IoT terminology used throughout this work specifically refers to healthcare edge devices (e.g., NVIDIA Jetson-class platforms) operating within hospital or clinic network environments as inference endpoints in a federated learning pipeline, consistent with the healthcare IoT edge deployment paradigm. The federated learning framework itself constitutes the networked, distributed system-level component, while individual edge devices serve as local inference and training nodes.

While our realistic federated deployment analysis demonstrates resilience under challenging conditions, the simulations remain controlled laboratory evaluations. Future work should include pilot deployments in actual multi-institutional healthcare networks with heterogeneous infrastructure to fully validate practical IoT suitability.

### 5.7 Broader impact and future applications

The proposed framework has broader implications beyond lung nodule analysis and could be adapted to other medical imaging tasks that involve approximately spherical or geometric structures. The success of attention-based approaches in recent work [17,32] suggests that our geometric attention framework could be beneficial for applications in breast cancer detection, brain tumor analysis, and liver lesion characterization.

The federated learning approach demonstrates the feasibility of privacy-preserving collaborative AI development in healthcare, potentially enabling the creation of global medical AI models that leverage diverse datasets while respecting patient privacy and regulatory requirements. This paradigm could accelerate medical AI development and improve healthcare outcomes worldwide, addressing challenges identified in recent comprehensive surveys [33,34].

The uncertainty quantification framework provides a template for developing clinically responsible AI systems that provide appropriate confidence estimates and acknowledge their limitations. This approach could help address the “black box” criticism of deep learning systems and facilitate regulatory approval and clinical adoption, representing a significant advancement over current approaches that lack uncertainty awareness [35,36].

## 6 Conclusion

This paper presents AGA-Net, a novel two-stage framework for lung nodule segmentation and malignancy classification that successfully integrates geometric constraints with attention mechanisms and uncertainty quantification in a federated learning environment. By incorporating the spherical nature of lung nodules through the Geometric-Constrained Attention Module and providing uncertainty-aware predictions through the Multi-Scale Uncertainty Quantification Network, our method achieves state-of-the-art performance across multiple benchmark datasets while addressing critical requirements for clinical deployment including privacy preservation, uncertainty quantification, and interpretability. The comprehensive evaluation demonstrates superior performance with Dice coefficients of 0.927 for segmentation and AUC of 0.951 for classification, along with excellent calibration quality and computational efficiency suitable for real-world healthcare IoT environments. The federated learning implementation enables collaborative model development while maintaining strict privacy protections, representing a significant advancement toward practical deployment of AI systems in distributed healthcare networks. The proposed framework addresses fundamental challenges in medical AI including uncertainty quantification, privacy preservation, and clinical interpretability, providing both technical innovation and practical clinical value through uncertainty-aware predictions that can guide radiological decision-making and improve patient outcomes in early lung cancer detection.

## Supporting information

S1 Appendix Parameter definitions. Table A1. Clarified parameter definitions and values.
(PDF)
