# Supplementary material for: Adaptive geometric-attention network for two-stage lung nodule segmentation and malignancy classification in federated healthcare IoT edge environments
Source: PLoS One. 2026 Jul 10;21(7):e0341096. doi: 10.1371/journal.pone.0341096 (PMC13354005; doi:10.1371/journal.pone.0341096)
Supplement: S1 Appendix — Table A1. Clarified parameter definitions and values. (PDF) [file pone.0341096.s001.pdf]

# Adaptive Geometric-Attention Network for Two-Stage Lung Nodule Segmentation and Malignancy Classification in Federated Healthcare IoT Edge Environments

Muhammad Sufyan[1], Jun Qian[1], Jianqiang Li[1], Azhar Imran[1], Fahad Sabah[1], Raheem Sarwar[2,*]

**1** College of Computer Science, Beijing University of Technology, Beijing, 100124, China
**2** OTEHM, Manchester Metropolitan University, Manchester, U.K.
*R.Sarwar@mmu.ac.uk.

# Appendix A: Parameter definitions

**Table A1.** Clarified parameter definitions and values.

| Parameter | Symbol | Value/Range | Description |
|---|---|---|---|
| Attention Spatial Weight | $\alpha_{att}$ | 0.4 (learnable) | Spatial attention fusion weight |
| Attention Channel Weight | $\beta_{att}$ | 0.3 (learnable) | Channel attention fusion weight |
| Attention Geometric Weight | $\gamma_{att}$ | 0.3 (learnable) | Geometric attention fusion weight |
| Radius Mean Weight | $\alpha_{rad}$ | 0.7 (learnable) | Adaptive radius mean component |
| Radius Std Weight | $\beta_{rad}$ | 0.2 (learnable) | Adaptive radius std component |
| Radius Bias | $\gamma_{rad}$ | 0.1 (learnable) | Adaptive radius bias component |
| Dirichlet Concentration | $\alpha_{dir}$ | 0.1–10.0 | Data heterogeneity parameter |
| Focal Loss Weight | $\alpha_{focal}$ | 0.25 | Focal loss balancing parameter |
| Privacy Noise Coefficient | $\alpha_{dp}$ | 0.023 | Privacy-utility trade-off parameter |
